# AXL expression reflects tumor-immune cell dynamics impacting outcome in non-small cell lung cancer patients treated with immune checkpoint inhibitor monotherapy

**DOI:** 10.3389/fimmu.2024.1444007

**Published:** 2024-08-21

**Authors:** Austin Rayford, Fabian Gärtner, Maria P. Ramnefjell, James B. Lorens, David R. Micklem, Marianne Aanerud, Agnete S. T. Engelsen

**Affiliations:** ^1^ Department of Biomedicine and Centre for Cancer Biomarkers, University of Bergen, Bergen, Norway; ^2^ Department of Thoracic Medicine, Haukeland University Hospital, Bergen, Norway; ^3^ Department of Clinical Science, Faculty of Medicine, University of Bergen, Bergen, Norway; ^4^ Department of Pathology, Haukeland University Hospital, Bergen, Norway; ^5^ Department of Clinical Medicine and Centre for Cancer Biomarkers, Faculty of Medicine, University of Bergen, Bergen, Norway; ^6^ BerGenBio ASA, Bergen, Norway

**Keywords:** NSCLC, biomarker, AXL receptor tyrosine kinase, immunotherapy resistance, tumor microenvironment

## Abstract

**Introduction:**

AXL receptor expression is proposed to confer immune-checkpoint inhibitor (ICI)-resistance in non-small cell lung cancer (NSCLC) patients. We sought to interrogate AXL expression in conjunction with mutational and tumor-microenvironmental features to uncover predictive mechanisms of resistance in ICI-treated NSCLC patients.

**Methods:**

Tumor samples from 111 NSCLC patients treated with ICI-monotherapy were analyzed by immunohistochemistry for tumor- and immune-AXL expression. Subsets of patients were analyzed by whole-exome sequencing (n = 44) and imaging mass cytometry (n = 14). Results were related to ICI-outcome measurements.

**Results:**

Tumor-cell AXL expression correlated with aggressive phenotypic features including reduced OS in patients treated with ICIs (*P* = 0.04) after chemotherapy progression, but conversely associated with improved disease control (*P* = 0.045) in ICI-treated, PD-L1 high first-line patients. AXL+ immune-cell infiltration correlated with total immune-cell infiltration and improved overall outcomes (PFS: *P* = 0.044, OS: *P* = 0.054). Tumor-cell AXL-upregulation showed enrichment in mutations associated with PD-L1-upregulation and ICI-response such as *MUC4* and *ZNF469*, as well as adverse mutations including *CSMD1* and *LRP1B* which associated with an immune-suppressed tumor phenotype and poor ICI prognosis particularly within chemotherapy-treated patients. Tumor mutational burden had no effect on ICI-outcomes and was associated with a lack of tumor-infiltrating immune cells. Spatial-immunophenotyping provided evidence that tumor-cell AXL-upregulation and adverse mutations modulate the tumor microenvironment in favor of infiltrating, activated neutrophils over anti-tumor immune-subsets including CD4 and CD8 T-cells.

**Conclusion:**

Tumor-cell AXL-upregulation correlated with distinct oncotypes and microenvironmental immune-profiles that define chemotherapy-induced mechanisms of ICI-resistance, which suggests the combination of AXL inhibitors with current chemoimmunotherapy regimens can benefit NSCLC patients.

## 1 Introduction

Treatment with immune-checkpoint inhibitors (ICIs) against programmed death protein 1 and programmed death-ligand 1 (PD-L1) have revolutionized therapy of patients with advanced non-small cell lung cancer (NSCLC). PD-L1 expression in lung cancer cells and tumor mutational burden (TMB) are the best available predictive biomarkers for ICI-response (1, 2) but have limited clinical utility. Even tumors with high TMB and PD-L1 tumor proportion score (TPS) can exhibit primary resistance to ICI-therapy (3) or develop acquired resistance after an initial response (4). Current research describing ICI-resistance mechanisms focuses on the impact of the peri-tumoral milieu of secreted factors and immune-, stromal-, and endothelial cells that comprise the tumor microenvironment (TME), and (epi-)genetic alterations in cancer cells that modulate TME composition (4–6). Somatic gene variants that produce neo-antigens which can be targeted by T-cells as well as specific immunogenic mutations leading to an inflamed TME are associated with ICI-responsiveness (7). Contrarily, mutations can also lead to an immune-evasive TME and promote ICI-resistance (8).

AXL is a member of the TAM (TYRO3, AXL, and MERTK) family of receptor tyrosine kinases and is expressed in many tumors including NSCLC (5, 6). AXL expression is associated with multiple processes in cancer progression, including invasive tumor growth, therapy resistance, a more migratory mesenchymal phenotype, and poor clinical outcomes (9–11), and is also expressed on tumor-associated immune and endothelial cells. AXL may contribute to an immune-evasive TME by impairing antigen presentation (12–14), increasing PD-L1 expression on cancer cells (15, 16), enabling intrinsic cancer cell-resistance to immune-mediated killing (17), and altering the cytokine profile in the TME to be more tumor-supportive (18). Unfavorable gene variants as well as epigenetic changes including AXL-upregulation are both proposed to act via conserved molecular programs such as epithelial-mesenchymal transition (EMT) (10, 19), wherein cancer cells gain stem-cell characteristics with migratory and immune-evasive properties (20) that thwart T-cell cytotoxicity and ICI treatment. AXL-inhibiting drugs might reverse EMT and sensitize resistant tumors to ICIs (21), and are currently being tested together with ICIs in NSCLC patients in clinical trials (NCT05469178, NCT03184571).

In our study, we investigated the clinical significance and correlation of AXL expression with mutational and microenvironmental features in ICI-treated NSCLC patients. The primary endpoint was that AXL expression on tumor and immune cells was associated with a negative impact on ICI prognosis. Surprisingly, we found differences in the predictive value of tumor- versus immune-AXL expression that correlated with distinct oncotypes and TME immune-profiles associated with ICI prognosis. In conclusion, our study provides comprehensive insight into features associated with response and resistance to ICI treatment.

## 2 Materials and methods

### 2.1 Patients and treatment

Patients with advanced NSCLC treated with ICI-monotherapy in routine clinical care at the Department of Thoracic Medicine at Haukeland University Hospital in Bergen, Norway between October 2016 and June 2019 were included in this retrospective study. Patients treated with ICIs in first-line (1L) received pembrolizumab and were all PD-L1 TPS ≥50% as a prerequisite for treatment with first line ICI-monotherapy. Patients treated with ICI-monotherapy in second and subsequent lines (2L) received either pembrolizumab, atezolizumab or nivolumab, and had PD-L1 TPS ≥1%. ICI treatment was only approved for PD-L1-positive patients in Norway at the time of this study, therefore no PD-L1-negative patients were included. Second line patients had relapsed or were refractory to prior chemotherapy. Primary chemoresistance was defined as radiologically-confirmed disease progression within 4 weeks after completing 2-4 cycles of 1L-chemotherapy.

Treatment was administered for up to two years and was discontinued if serious toxicities occurred or upon radiological confirmation of progressive disease. The censoring-date for registration of clinical and survival data was February 1^st^ 2021. Information from the electronic health record was used to register patient-, cancer-, and treatment-related data. Written informed consent was obtained from patients alive before inclusion. Deceased patients were included posthumously without consent. The regional committee for medical and health research ethics approved the study (REK 2019-45569).

### 2.2 Tumor samples

The total number of ICI treated patients in the period October 2016 - June 2019 was 157. Of these, 121 patients had tumor samples which could be stained with anti-AXL antibodies, 10 samples had to be rejected due to insufficient material when assessed by the pathologist, therefore the total number in the AXL IHC cohort is 111. Patient characteristics like age, gender, performance status, or metastatic burden did not differ between the whole ICI treated cohort of 157 patients described by Gärtner et al. (22), and the AXL IHC cohort of 111 patients. Just a few patients of the AXL IHC cohort (4 out of 111) were not evaluated for AXL-expression on immune cells due to insufficient material with scarce stroma in the samples. Tumor samples were obtained during routine clinical care prior to ICI-therapy either via biopsy of the primary lung cancer (n=67) or distant metastases (n=27), or via prior surgical resection of the lung cancer (n=17). Tumor samples were formalin-fixed and paraffin-embedded (FFPE), and sections were subjected to routine PD-L1 immunohistochemistry (IHC) staining using the Ventana SP263 kit. PD-L1 TPS was routinely scored by clinical pathologists as either low (1-49%) or high (≥50%). Non-squamous tumors were routinely analyzed for EGFR mutations and ALK- and ROS-1 translocations. Samples obtained prior to chemotherapy or radiotherapy (RT) were defined as pretreatment, while samples obtained after initial lines of chemotherapy or RT were defined as posttreatment.

### 2.3 Immunohistochemical evaluation of AXL expression

AXL-IHC staining of FFPE-tumor sections was performed on an automated Ventana tissue stainer (anti-AXL clone 7E10) and scored according to study-specific guidelines by a trained pathologist at Roche Tissue Diagnostics (Tucson, Arizona, USA). Tumor-cell AXL expression (tAXL) was quantified via “Histo-score” (Hscore), which ranges from 0-300 and represents the sum of the percentages of cancer-cell staining at each intensity level multiplied by the cytoplasmic staining intensity (0 = no staining, 1 = weak-staining, 2 = moderate-staining, and 3 = strong-staining). Sections were scored for the abundance of total and AXL-expressing tumor-infiltrating immune cells (Total IC and AXL IC, respectively) as a percentage of all cells within the tumor area. Patient groups stratified by predefined cutpoints for AXL Hscore, AXL IC and total IC were compared by survival analysis to test for association of these metrics with ICI outcomes. All Kaplan-Meier plots and the optimal AXL Hscore-cutpoint (lowest p-value) for tAXL-high 2L-patients were generated using the survminer package in R. Representative AXL IHC images were generated from digital slide scans using QuPath software (23).

### 2.4 Whole-exome sequencing

FFPE-tumor samples with sufficient tissue material for WES (n=44) were analyzed by Almac Diagnostics (Craigavon, UK) on an Illumina NovaSeq 6000 instrument. Alignment, quality-control, variant-calling, filtering of germline variants and FFPE-artifacts, and calculation of tumor mutational burden (TMB) and microsatellite instability (MSI) scores were performed in the proprietary Almac WES Software Suite using the “tumor-only (TO)” pipeline, which does not require matched normal tissue. MSI score was derived from an algorithmic estimation of somatic MSI sites relative to the total number of MSI sites detected in each patient, with >20% somatic MSI sites defining MSI-high tumors (24). Annotated somatic variants for each patient were converted to a single maf file using vcf2maf (25) and imported into R for analysis using the maftools R package. For mutation enrichment analysis, only genes mutated in ≥4 patients in either group were included.

### 2.5 Imaging mass cytometry staining

FFPE-surgical resection specimens (n=16) were core-sampled in triplicate at the tumor margin into a tissue microarray (TMA) that was sectioned and stained as described previously (26) with an optimized panel of heavy metal-conjugated antibodies (**Supplementary Table 6**). Briefly, slides were deparaffinized in xylene and rehydrated in descending grades of ethanol before being subjected to antigen retrieval (Dako S2637, pH 9) for 30 min at 95°C and cooled for 20 min. After washing with PBS, the TMA area was encircled with a hydrophobic pen and blocked with 5% BSA for 1h at RT. Next, primary mouse and rabbit antibodies against AXL intracellular and extracellular domains (ICD and ECD, respectively) were incubated for 1h at 36°C followed by three PBS-T washes and incubation with metal-conjugated secondary anti-mouse and anti-rabbit antibodies for 30 min at RT. After washing, sections were incubated with a cocktail of the remaining 38 metal-conjugated antibodies overnight at 4°C, washed again, stained with Iridium DNA-intercalator (Fluidigm #201192B), washed 2X with PBS, dipped briefly in deionized water and allowed to air dry before ablating the tissue on a Hyperion Imaging Mass Cytometer (Standard Biotools, Inc.).

### 2.6 IMC data preprocessing

The resulting 42-plex IMC images for each tissue core were visually examined for marker staining and tissue quality, resulting in the exclusion of CCR7, CD123 and NKp46 markers and 9 cores that contained little-to-no tumor tissue (including all three cores from two patients, resulting in a final analysis set of 14 patients). Single-cell segmentation masks were generated for each image using the Steinbock preprocessing pipeline (27), using aggregated images of the two Ir-DNA intercalator channels and the three cell segmentation marker channels as the nucleus and cytoplasm inputs to DeepCell, respectively. A binary tumor segmentation mask was generated by training a pixel classifier in Ilastik (v1.3.3) on pan-cytokeratin, E-cadherin, and Ir-DNA channel images to classify pixels as tumor, stroma or background, feeding the resulting probability images into CellProfiler (v4.2.1) and applying a binary threshold of 0.5 on the tumor prediction channel.

Mean marker-intensity and spatial information for each cell was then extracted and imported into R for preprocessing, following the IMC Data Analysis Workflow (27). Metal signal spillover correction was performed using the CATALYST package and a previously-generated spillover matrix (28), and arcsine-transformed (cofactor 1). In order to account for strong variations in overall marker staining intensity observed between tissue samples, sample integration (a.k.a. batch correction, where each tumor sample is treated as a unique batch) was performed; first using the Harmony package to generate batch-corrected UMAP-reduced dimensions of type marker expression per cell (**Supplementary Figure 7A**), and later using the Seurat package to batch-correct and scale the single-cell expression levels of all markers. The reasoning for employing two different sample integration approaches was that Seurat tended to overcorrect the data relative to Harmony, resulting in poorly-differentiated clusters, while Harmony uses PCA-reduced dimensions as input and does not directly batch-correct the marker expression data itself, which was necessary for reliable comparison of state marker expression levels between samples.

### 2.7 Annotation of IMC cell-types

Each cell was assigned a cell-type label using a combination of unsupervised clustering plus manual merging and gating guided by visual confirmation of cell clusters overlaid on staining images. Clustering was performed on Harmony-corrected, UMAP-reduced dimensions of type marker expression using SNN graph-based clustering (k=7) and the resulting 22 clusters were manually merged and annotated. Further adjustment and splitting of clusters was performed by overlaying cells on staining images using the Cytomapper package and manually thresholding on 1-2 markers, resulting in the generation of CD15+HIF1a+ neutrophil, CD15-HIF1a+ (other/non-neutrophil), Treg (FOXP3+) and Clec9A+ clusters. Finally, the myeloid cluster was sub-clustered using the same parameters on Seurat-corrected expression levels of only myeloid type markers, generating distinct monocyte-derived dendritic cell (MoDC) (CD11c+) and CD163+ subpopulations. All supervised steps in the annotation pipeline were blinded to patient status. Cell-type abundance and mean state marker expression levels (Seurat-corrected) were then calculated for each core for all cells and cells residing within the tumor mask (intratumoral cells) and used as independent data points in statistical tests between groups. One patient with undifferentiated histology had a non-compartmentalized tumor morphology and was excluded from analyses of intratumoral cells.

### 2.8 Statistics

Descriptive models were used to characterize the sample. All variables with non-normal distributions were analyzed by nonparametric tests including Mann-Whitney U-test and Spearman’s Rho correlation. Variables examined by cross-tabulation were compared by Chi-square tests. Significance was defined as a 2-sided p ≤ 0.05. Stratified patient groups were compared by Kaplan-Meier plots and log-rank test, while cox proportional-hazard models were used to test for association of continuous or multiple variables with survival. Data management and statistical analysis were performed with IBM SPSS statistics version 26 and R version 4.2.3.

## 3 Results

### 3.1 AXL expression in tumor cells associates with PD-L1-upregulation, non-squamous histology and an aggressive cancer phenotype

Tumor samples from 111 NSCLC patients treated with ICI monotherapy in first line (1L) or after one-or-more lines of chemotherapy (2L) (**Supplementary Table 1**) were evaluated for AXL expression by immunohistochemistry (IHC). Approximately half (51%) of all patients had no detectable tAXL expression (AXL Hscore=0), with no differences between pre- and posttreatment tumor samples. Representative IHC images of squamous and non-squamous tAXL high and -low samples are presented in **Supplementary Figures 1A–C**.

**Figure 1:**
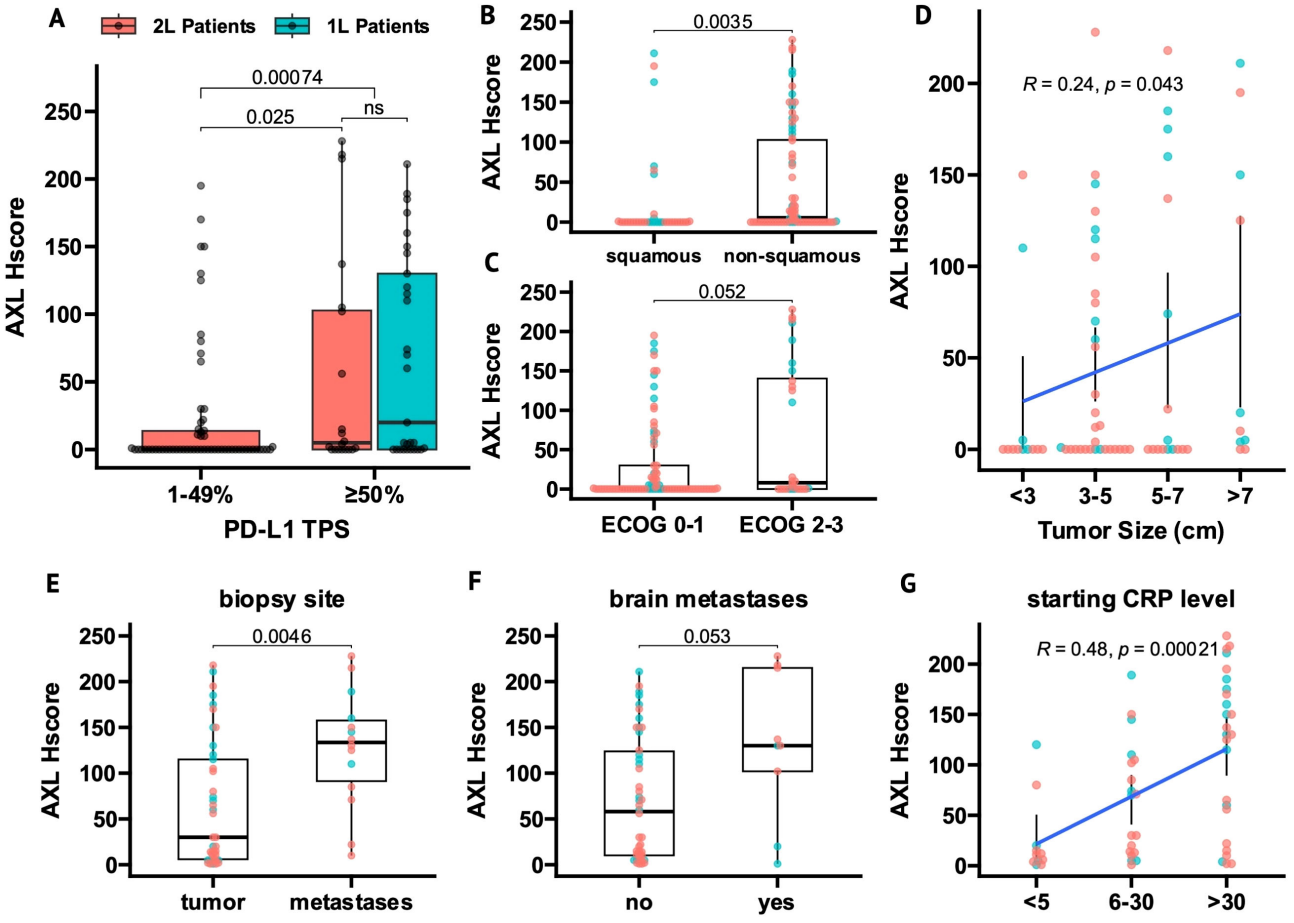
AXL expression in tumor cells (tAXL) associates with PD-L1-upregulation, non-squamous histology and an aggressive cancer phenotype. **(A)** Boxplot of tAXL expression (AXL Hscore) in first-line (1L, cyan) and beyond first-line (2L, red) tumor samples split by PD-L1 TPS (low, 1-49%; high, ≥50%). **(B, C)** Boxplots of AXL Hscores in ECOG PS 2-3 vs 0-1 patients **(B)** and in non-squamous vs squamous tumors **(C)**. **(D)**, Scatter plot of AXL Hscore vs baseline tumor size as an ordinal variable. **(E–G)** In tAXL-positive tumors, boxplots show higher AXL Hscores in metastatic versus primary tumor samples **(E)** and in patients with vs without brain metastases **(F)**, and scatter plot shows AXL Hscore correlated with starting CRP level as an ordinal variable **(G)**. p-values in boxplots and scatterplots from Mann-Whitney *U* test and Spearman correlation, respectively.

AXL Hscores were higher in tumors with high PD-L1 TPS (≥50%) vs low PD-L1 TPS (1-49%), overall (*P* = 0.00074, **Figure 1A**), and within 2L-patients (*P* = 0.025, **Figure 1A**). In PD-L1-high tumors, AXL Hscores were similar between 1L- and 2L-patients (**Figure 1A**).

Non-squamous tumors had higher AXL Hscores than squamous tumors overall (*P* = 0.0035, **Figure 1B**) and in 2L-patients (*P* = 0.006, **Supplementary Figure 1A**), though not significantly in 1L patients (*P* = 0.13, **Supplementary Figure 1D**).

tAXL expression correlated with features of a more aggressive cancer phenotype. AXL Hscore trended higher in patients with ECOG PS 2-3 vs ECOG PS 0-1 (P = 0.052, **Figure 1C**) and positively correlated with initial tumor size (P = 0.043, **Figure 1D**). When stratifying patients by either metastatic versus primary sampling-site, presence of brain metastases or baseline C-reactive protein (CRP) levels, the proportion of tAXL-negative samples in each subgroup were nearly identical (**Supplementary Figures 1E–G**). However, in tAXL-positive samples, AXL Hscores were higher in distant metastases versus primary tumors (*P* = 0.0046, **Figure 1E**), trended higher in patients with- versus without brain metastases (*P* = 0.053, **Figure 1F**) and correlated with increasing CRP-level (*P* = 0.0002, **Figure 1G**).

### 3.2 Tumor AXL expression is a negative-prognostic in 2L-ICI patients and is associated with chemoresistance

**Figure 2:**
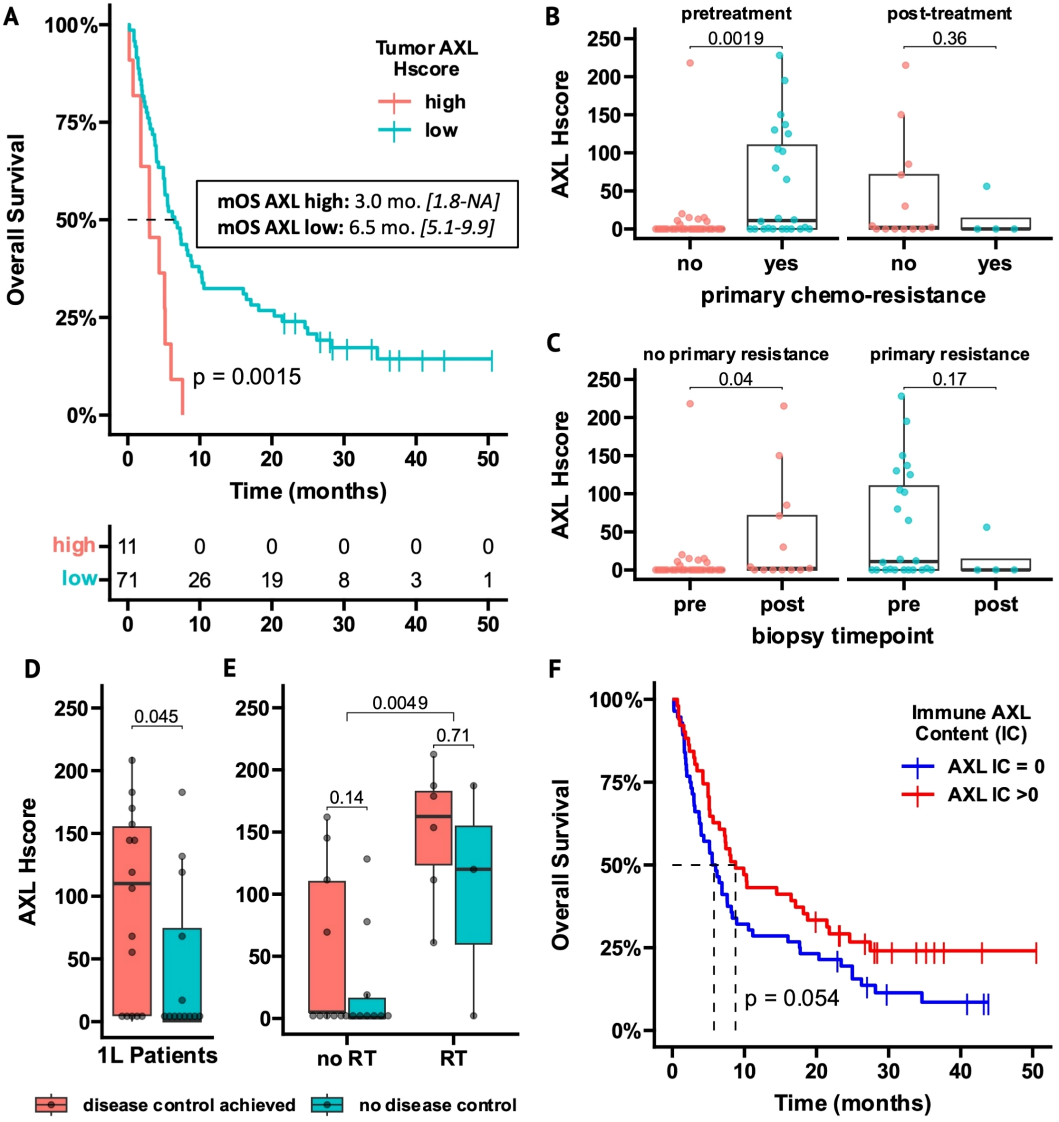
AXL expression on tumor cells and tumor-infiltrating immune cells is related to outcome measurements in ICI-treated NSCLC patients. **(A)** Overall Survival (OS) of 2L-ICI patients stratified by high- vs low tAXL expression using an optimal cutpoint of Hscore >105 (95% confidence-interval in brackets). **(B, C)** Boxplots of AXL Hscores in tumor samples obtained before or after 1L-chemotherapy, stratified by primary chemoresistance **(B)** or biopsy timepoint **(C)**. **(D, E)** Boxplots of AXL Hscores in 1L-patients stratified by disease control **(D)** and within 1L-subgroups with or without radiotherapy (RT) before/during ICI treatment **(E)**. **(F)** OS of all AXL IC-evaluable patients (n = 107) stratified by presence of AXL+ tumor-infiltrating immune cells (AXL IC>0). p-values in boxplots and Kaplan-Meier plots from Mann-Whitney *U* test and log-rank test, respectively.

Overall, no significant differences in DCR, PFS or OS were observed between patients with or without tAXL expression (**Supplementary Table 2**). In 2L-patients, however, higher tAXL expression was associated with shorter OS as a continuous variable (HR 1.004, *P* = 0.04). The optimal AXL Hscore cutpoint for tAXL was 105, representing the cut point with the most significant difference in OS (lowest p value) in 2L patients. Therefore, we used this cut point to divide between high and low in the analysis. The mOS was 3 months vs 6.5 months for 2L-patients with high vs low tAXL expression, respectively (*P* = 0.0015, **Figure 2A**).

In pretreatment tumor samples, 2L-patients with primary chemoresistance had higher tAXL expression than patients without resistance (*P* = 0.0019, **Figure 2B**). Furthermore, in patients without primary chemoresistance, tAXL expression was higher in samples taken after chemotherapy progression vs before treatment (*P* = 0.04, **Figure 2C**). This suggests that pre-existing tAXL expression promotes primary chemoresistance and is also upregulated during treatment to confer adaptive resistance.

tAXL expression was higher (*P* = 0.008) in 2L-patients starting ICIs within 6 months after chemotherapy discontinuation due to early progression (n=59, 72%). These patients experienced less benefit from ICI treatment (mOS 5 vs 10 months, *P* = 0.013; mPFS 2 vs 7 months, *P* = 0.014, data not shown), further supporting that tAXL expression elicits a treatment-resistant cancer phenotype.

Conversely, in 1L-patients, a trend towards better DCRs and longer PFS was seen in tAXL-positive patients (**Supplementary Table 2**). Furthermore, 1L-patients that achieved disease control (DC) on ICI treatment had higher AXL Hscores than patients with progressive disease (*P* = 0.045, **Figure 2D**). We found that 1L-patients who required radiotherapy (RT) to achieve local tumor control in the thorax or to treat symptomatic bone metastases had higher AXL Hscores at diagnosis (*P* = 0.0049, **Figure 2E**). Differences in tAXL expression between 1L-patients with and without DC were no longer significant within subgroups stratified by RT treatment (**Figure 2E**), but the number of patients within the subgroups (RT-treated: n=12) was small.

### 3.3 AXL+ infiltrating immune cells reflect total immune-infiltration and are a positive ICI-prognostic

Approximately half of all patients (52%) lacked detectable AXL-expressing intratumoral immune cells (AXL IC=0, **Supplementary Table 1**). Representative IHC images of squamous and non-squamous AXL-IC high and -low samples are presented in **Supplementary Figures 2A, B**. Most AXL IC-negative patients also had few if any total infiltrating immune cells (total IC<2%) and AXL IC was strongly correlated with total IC (Spearman R=0.77, p < 0.001, **Supplementary Figure 2C**). Therefore, AXL IC-score can be roughly interpreted as a surrogate measurement of total immune-infiltration. Overall, AXL IC-negative patients had shorter OS (*P* = 0.054) and PFS (*P* = 0.044, **Figure 2F** **/** **Supplementary Figure 2D**) and similar results were observed for patients with total IC<2% (**Supplementary Table 2**), indicating that the presence of tumor-infiltrating immune cells, whether measured via simple morphological assessment or staining of the AXL+ immune-subset, associated with improved outcomes.

### 3.4 Gene mutations correlate with tumor features and ICI outcomes

**Figure 3:**
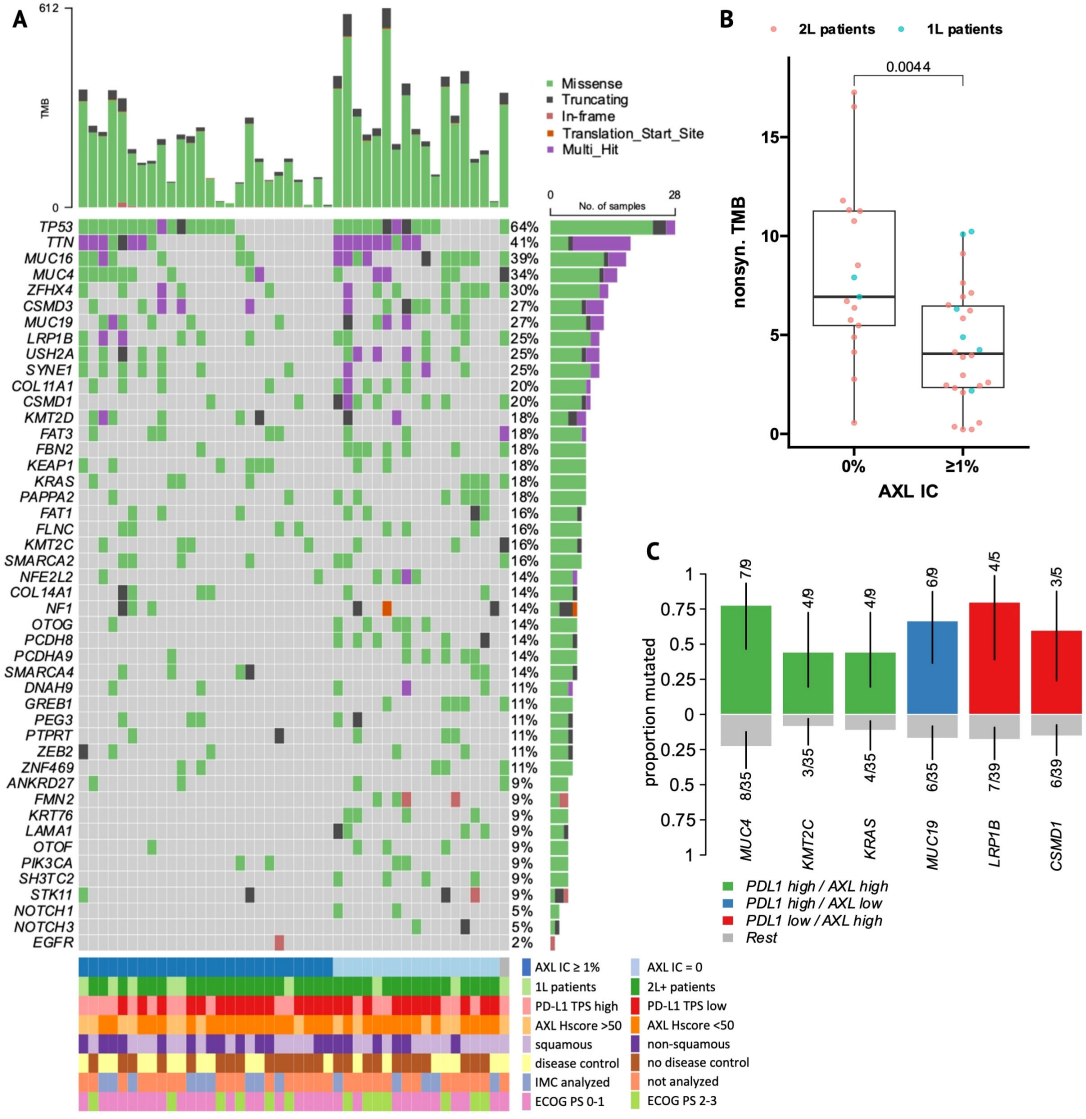
Mutational landscape of NSCLC patients. **(A)** Oncoplot of the top 10 most frequently-mutated genes and relevant mutations from the literature and current analysis. Patient columns are stratified by presence versus absence of AXL+ infiltrating immune cells. **(B)** Boxplot of non-synonymous tumor mutational burden (TMB, mutations per megabase) in AXL IC-negative versus -positive patients. **(C)** Enrichment plot of gene mutations that occur significantly more frequently (X^2^ p<0.05) in PDL1 TPS-high, AXL Hscore-high (Hscore >50) or double-positive patient groups.

Whole-exome sequencing (WES) was performed on 44 tumor samples from the whole AXL-IHC cohort, with a similar distribution of clinical and molecular features (**Supplementary Table 1**). After variant annotation and filtering, 9438 protein-altering mutations were found in 6142 genes, with a median of 207 mutations per patient. The 10 most frequently-mutated genes were *TP53, TTN, MUC16, MUC4, ZFHX4, CSMD3, MUC19, LRP1B, SYNE1*, and *USH2A* (**Figure 3A**). Tumor mutation burden (TMB) and microsatellite instability (MSI) scores were not correlated (**Supplementary Figure 4A**). TMB had no effect on ICI outcomes overall nor within 1L-/2L- or histology subgroups (**Supplementary Figure 3**), but strikingly, patients lacking AXL+ immune cells had higher TMB (*P* = 0.004, **Figure 3B**), with similar results observed for Total IC<2% (**Supplementary Figure 4B**). This trend remained significant in 2L-patients and was still visible within histology subgroups (**Supplementary Figures 4C, D**). No tumors were MSI-high (MSI range~2-4%). When analyzed as a continuous variable, MSI score showed no effect on ICI outcomes overall, but within 2L-patients, higher MSI score was associated with longer PFS and OS (**Supplementary Figure 3**). 1L-patients showed a strong opposite correlation but the number of patients was small (n=9).

Enrichment analysis was performed to identify genes and pathways more frequently-mutated within clinical and biomarker subgroups (**Supplementary Figure 5**). *KRAS* was exclusively-mutated in non-squamous tumors, whereas *FMN2* and *PIK3CA* were exclusively-mutated in squamous tumors. When grouping mutations by affected signaling pathways, squamous tumors were also enriched in NRF2 and PI3K pathway mutations, while AXL IC-negative patients were highly-enriched in mutations in NOTCH-signaling genes (*P* = 0.0016).

**Figure 4:**
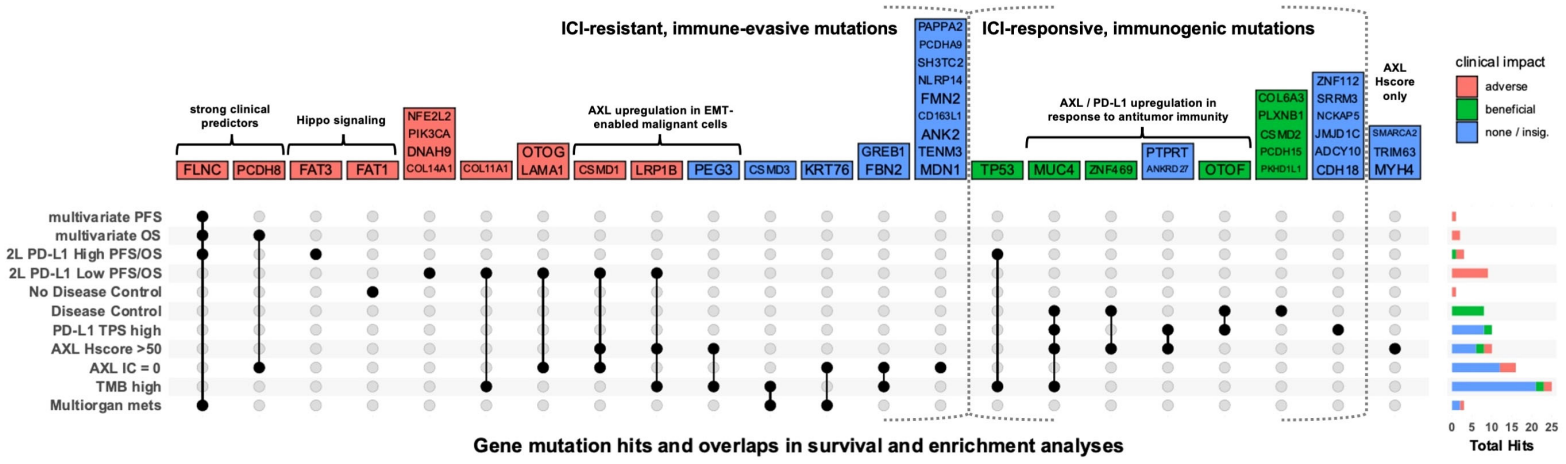
Association of gene mutations with ICI outcomes and biomarker status. UpSet plot showing gene mutations that were significantly (p<0.05) enriched in patient groups or associated with PFS or OS in multivariate analysis of all WES patients or univariate analysis within 2L-PD-L1 TPS-high and -low subgroups. Each column represents a set of gene mutations that was significant in a specific combination of one-or-more survival or enrichment analyses. Color represents whether a mutation was associated with improved PFS, OS, or disease control (green); reduced PFS, OS, no disease control (red); or only associated with biomarkers or metastases in more than one organ system (multiorgan mets) (blue). Right barplot shows the total number of significant gene mutations (hits) in each analysis. For all biomarker groups (PD-L1, AXL Hscore, AXL IC, and TMB), there were no enriched gene mutations in the opposing group (e.g. PD-L1 TPS-low).

To identify gene mutations associated with ICI outcomes, genes mutated in ≥5 patients were screened by Kaplan-Meier survival analysis. Mutations in nine and seven genes were associated with PFS and OS, respectively (**Supplementary Table 4**). Among them, *MUC4*, *FRAS1*, and *ZEB2* mutations were associated with longer PFS and/or OS. Conversely, mutations in other genes including *FLNC*, *PCDH8*, *FAT1*, *COL11A1*, *CUBN* and *TENM1* were associated with shorter PFS and/or OS. Significant genes by univariate analysis were introduced into multivariate cox regression models adjusted for significant clinical covariates (**Supplementary Figure 6**). *FLNC*-mutation was an independent predictor of both poor OS (HR = 6.08, 95% CI [1.3-28], *P* = 0.02) and PFS (HR = 6.03, 95% CI [1.5-29], *P* = 0.01), while *PCDH8*-mutation predicted poor OS (HR = 5.31, 95% CI [1.5-18], *P* = 0.009). Furthermore, we performed survival analysis within 2L-PD-L1 TPS-high and -low subgroups to identify mutations that differentially affected ICI responses within each group (**Figure 4**, **Supplementary Table 5**).

### 3.5 Tumors with high tAXL expression are enriched in both ICI-predictive and poor-prognostic mutations

WES-patients with high tAXL expression (above mean, Hscore>50) tended to have longer PFS (**Supplementary Table 3**), similar to 1L-patients in the whole AXL-IHC cohort. Likewise, the strong association between high PD-L1 TPS and AXL Hscore remained significant in the WES subset (**Supplementary Figure 4E**). Tumors with high tAXL and PD-L1 expression were enriched in MUC4 mutations (**Figure 3C**), which were associated with longer PFS (**Supplementary Table 4**) and enriched in DC-patients (**Figure 4**). These tAXL/PD-L1 double-positive patients were also enriched in *KRAS* and *KMT2C* mutations (**Figure 3C**); both positive-predictive markers for ICIs in NSCLC (29, 30). *KRAS*-mutation status had no effect on ICI outcomes in this cohort, even within non-squamous patients where *KRAS* was exclusively-mutated. *KMT2C* mutations, however, were enriched in partial-responders to ICIs (**Supplementary Figure 5N**). Furthermore, we identified a cluster of pairwise-enriched gene mutations between AXL-high, PD-L1-high and DC-patients that consisted of *OTOF*, *ZNF469*, *PTPRT*, and *ANKRD27* (**Figure 4**).

Conversely, 2L WES-patients with high AXL Hscores had numerically-shorter OS (**Supplementary Table 3**), in line with the whole AXL-IHC cohort. The majority of 2L tAXL-high WES-patients were PD-L1-low, suggesting a different role for tAXL expression in 2L-patients independent of PD-L1-upregulation. These tAXL-high/PD-L1-low patients were enriched in *CSMD1* and *LRP1B* mutations (**Figure 3C**), which were each associated with poor OS within 2L-PD-L1-low patients (**Figure 4**). *CSMD1* mutations were also enriched in immune-cold, AXL IC-negative patients.

### 3.6 Imaging mass cytometry captures the tumor-immune landscape

To investigate tumor-immune dynamics, we performed 40-plex IMC on a subset of 14 patients (**Supplementary Table 1**) with surgical resection specimens that were core-sampled in triplicate into a tissue microarray (TMA).

**Figure 5:**
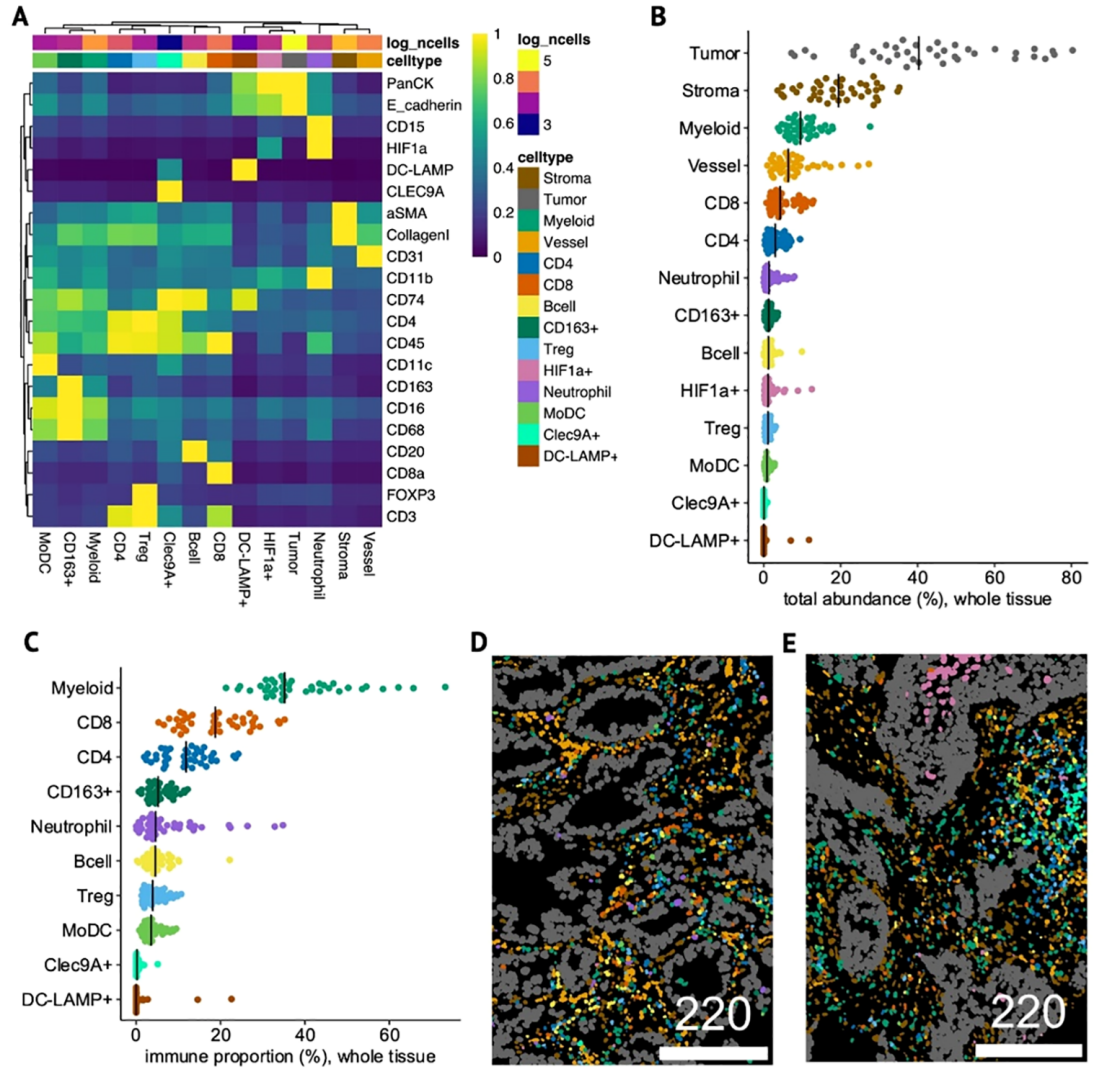
Identity and composition of cell-types in NSCLC tumor samples. **(A)** Heatmap of mean phenotypic marker expression (min-max-scaled) in each of the 14 annotated cell-types detected by IMC. **(B, C)** Dotplots showing total abundance of each cell-type relative to all cells (whole-tissue) **(B)** and proportion of each immune cell-type relative to only immune cells in whole-tissue **(C)**. **(D, E)** Single-cell segmented IMC images colored by cell-type in a non-squamous, ICI OS-low patient with high tAXL expression (Hscore=170) **(D)** and in a squamous, ICI OS-high, AXL Hscore-negative patient **(E)**. Scale bars in μm.

A total of 237,719 cells were detected and assigned to 14 distinct cell-types (**Supplementary Figure 7A**) including tumor, stromal and vessel cells and various immune lineages (**Figure 5A**).

To aid in the identification of neutrophils, HIF1a was included as a type-marker, resulting in a single HIF1a+ cluster that was split into CD15+ neutrophils and other HIF1a+ tumor and stromal cells. We also identified rare Clec9A+ and DC-LAMP+ cells, markers for cDC1 and mregDC dendritic-cell subsets, respectively (31). Considering that some Clec9A+ cells were also DC-LAMP+, the Clec9A+ cluster likely represents cDC1 on a spectrum with mregDCs, whereas the DC-LAMP+ cluster, which was pan-cytokeratin/E-cadherin+, may comprise either mregDC-infiltrated tumor cells, DC-LAMP+ tumor-associated type-II pneumocytes (32), or some combination of the two. Both clusters were distinct from the broader CD11c+ cluster, termed monocyte-derived dendritic cells (MoDCs) due to their high abundance and expression of other myeloid markers including CD16/CD68.

For each TMA-core, we quantified the relative total abundances of each cell-type across the whole tissue area (**Figure 5B**) and within the tumor mask (intratumoral, **Supplementary Figure 7B**). The total proportion of tumor cells varied significantly between cores, but when subsetting to intratumoral cells (denoted as “*i-*”), tumor cells constituted the vast majority of *i*-cells in most cases, effectively normalizing for tumor area. To account for general variations in overall immune-cell abundance and infiltration, we calculated the proportion of each immune cell-type relative to only immune cells (immune-proportion) for whole-tissue (**Figure 5C**) and *i*-cells (**Supplementary Figure 7C**). Both overall and within the tumor compartment, the myeloid cluster dominated the immune landscape, followed by CD8 T-cells. CD4 T-cells and CD163+ macrophages were the next highest in whole-tissue, but within tumors, *i-*neutrophils and *i*-MoDCs were more prevalent.

### 3.7 Immune dynamics associate with histology and therapeutic outcomes

**Figure 6:**
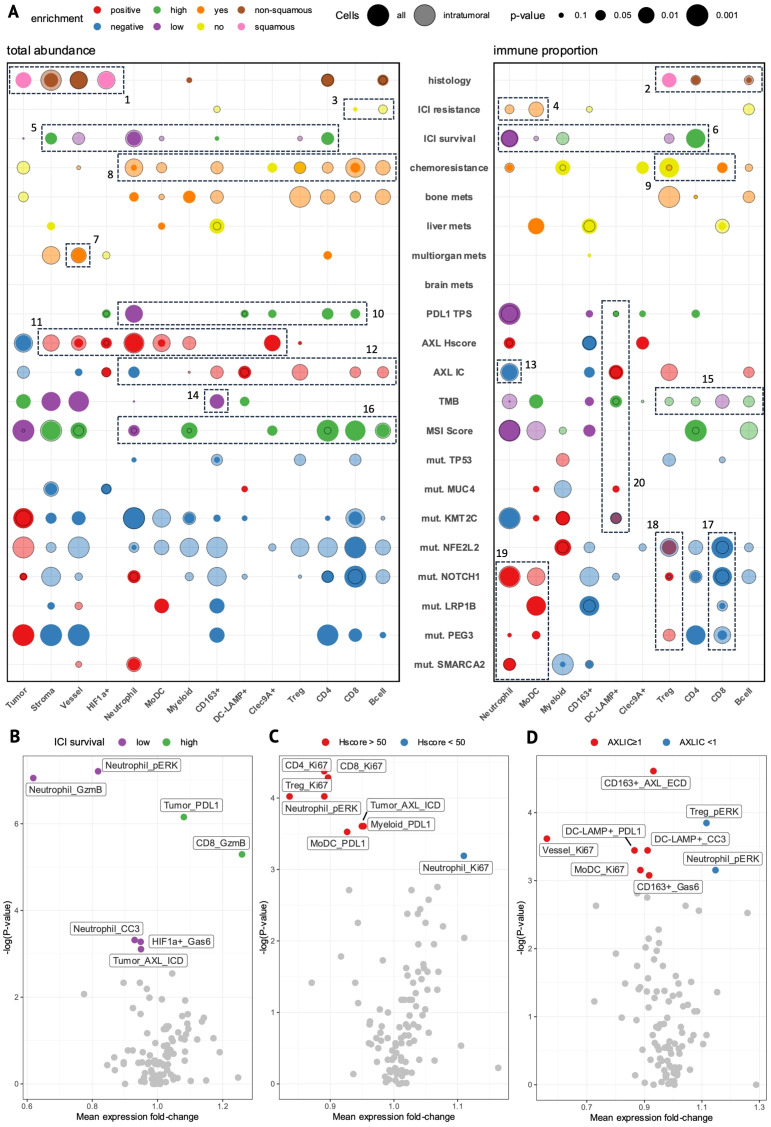
The cell-type composition and functional state of specific cell types associates with clinical and molecular features. **(A)** Bubble-plot showing enrichment of cell-type abundance (left) and immune proportion (right) of cell-types in tumor cores stratified by clinical or molecular features. Circle size represents the degree of significance and color represents the group in which the enrichment occurs. Solid circles represent enrichment in the whole tissue area (all cells) while transparent circles represent enrichment within the tumor boundary (intratumoral cells). **(B–D)** Volcano plots of significance (p-value) versus mean protein expression (fold-change) show differentially-expressed state markers within each cell-type (cutoff: p<0.05, Mann-Whitney) between tumor cores stratified by ICI survival-low (purple) versus -high (green) **(B)**, AXL Hscore-high (Hscore >50, red) versus -low (Hscore <50, blue) **(C)**, and AXL IC-positive (AXLIC ≥1, red) versus -negative (AXLIC <1, blue) **(D)**. **(B,D)** generated from intratumoral cells; **(C)** generated from all cells.

To observe the interplay between TME composition and clinical and molecular features, we performed rank-based comparisons of cell-type abundance and immune-proportions for both total and *i*-cells in each core between patient groups (**Figure 6A**).

The IMC subset contained mostly pretreatment tumor samples from 2L-patients (n= 12/14, **Supplementary Table 1**) with evenly-mixed histologies (**Supplementary Table 1**). Intratumoral stromal and vessel cells were increased in non-squamous tumors (**Figure 6A**, Box 1), indicating more diffuse growth patterns (**Figure 5D**), whereas squamous tumors had a corresponding enrichment of tumor and *i*-HIF1a+ cells reminiscent of solid tumor architecture with insufficient tumor oxygenation (**Figure 5E**). We also observed higher CD4 T- and B-cell immune-proportions in non-squamous tumors versus increased regulatory T-cell (Treg) proportions in squamous tumors (**Figure 6A**, Box 2), consistent with previous reports (33).

Although there were few associations between ICI-response and cell-type abundance, we did observe modest increases in CD8 T- and *i*-B-cells in DC-patients (no ICI-resistance, **Figure 6**, Box 3) with a marked decrease in *i*-neutrophil and *i*-MoDC immune-proportions (**Figure 6**, Box 4). When splitting patients on OS into ICI survival-low (<15 months) vs high (>15 months, n=6) groups, however, we found stronger evidence of early immune dynamics impacting long-term ICI outcomes. Here, we observed enrichment of neutrophils and *i*-vessel cells in OS-low patients, whereas CD4 and stromal cells were enriched in OS-high patients (**Figure 6**, Box 5). The inverse prognostic relationship between CD4 T-cells and neutrophils was even more apparent as an immune-proportion, where *i*-Tregs were also enriched in OS-low patients (**Figure 6**, Box 6). This supports the essential role of CD4 T-cells in promoting sustained anti-tumor immune responses as well as the immunosuppressive role of Tregs and neutrophils. Furthermore, *i*-vessel cells were also enriched in patients with multi-organ metastases (**Figure 6**, Box 7), suggesting that tumor vascularization may facilitate metastasis and impact ICI prognosis independently of immune dynamics.

In chemoresistant patients, we observed broadly-elevated immune-infiltration (**Figure 6**, Box 8), with increased CD8 and reduced Treg immune-proportions (**Figure 6**, Box 9). Taken together, chemoresistant patients showed evidence of active anti-tumor immune responses. Patients with bone metastases at ICI-start showed a near-identical enrichment pattern, suggesting that tumor-immune targeting elicits a chemoresistant, metastatic tumor phenotype.

### 3.8 ICI-predictive biomarkers display distinct immunophenotypes

IMC-patients with high AXL Hscores (>50) tended to have low PD-L1 TPS and poor OS, capturing the subset of 2L-patients where tAXL expression had a poor-prognostic effect. PD-L1 TPS-high patients showed a similarly-strong reduction of neutrophils and increase in CD4 T-cells as ICI OS-high patients, while also having modest increases in CD8, Clec9A+ and DC-LAMP+ cells (**Figure 6**, Box 10).

tAXL-positive tumors had higher intratumoral densities of MoDCs, neutrophils, and stromal, vessel and myeloid cells (**Figure 6**, Box 11), suggesting that tAXL positive tumors foster a more immunosuppressive environment by recruiting inflammatory mediators.

AXL IC-positive tumors had a near-opposite immune-infiltration profile (**Figure 6**, Box 12) including CD8 and DC-LAMP+ cells, Tregs, and CD163+ macrophages, despite only moderate overlap with tAXL -negativity (**Supplementary Figure 7D**). This was accompanied by a strong decrease in both total- and *i*-neutrophil proportions (**Figure 6**, Box 13). The increased abundance of CD8 T-cells, a key determinant of ICI efficacy, along with the reduction in immunosuppressive neutrophils, supports the association of AXL IC-positivity with improved ICI outcomes observed in the whole AXL-IHC cohort.

TMB-high patients (above-median) showed no reduction in immune-cell abundances except CD163+ macrophages (**Figure 6**, Box 14) despite showing a general lack of immune-infiltration in IHC. When comparing immune-proportions, however, we found a modest increase in all *i*-lymphocytes except *i*-CD8 T-cells, which were conversely enriched in TMB-low tumors (**Figure 6**, Box 15). Meanwhile, patients with above-median MSI scores had the strongest collective enrichment of total CD4, CD8, and B-cells of any group comparison and a corresponding reduction in neutrophils (**Figure 6**, Box 16), supporting the association of higher MSI score with improved 2L-ICI prognosis.

Patients with ICI-resistant, immune-evasive mutations had lower immune-proportions of i-CD8 T-cells (**Figure 6**, Box 17). Moreover, these mutations, which were evenly-distributed amongst half (7/14) of IMC-patients (**Supplementary Figure 7E**), showed increased immune-proportions of neutrophils, MoDCs and Tregs (**Figure 6**, Boxes 18, 19), indicative of an immunosuppressive TME in which MoDCs may also play a role.

Patients with *MUC4* and *KMT2C* mutations, both positive-predictive markers, showed no enrichment of anti-tumor immune subsets with the exception of DC-LAMP+ cells, which were also the only immune-enriched cell-type across AXL IC-positive, PDL1 TPS-high and TMB-high patients (**Figure 6**, Box 20), suggesting that the DC-LAMP+ cluster captures immune-activating mregDCs. The lack of stronger evidence for anti-tumor immunity in tumors with these mutations is likely due to their high co-mutation rate with adverse mutations in IMC-patients (**Supplementary Figure 7E**).

### 3.9 Differential expression identifies functional substates associated with ICI prognosis and tumor/immune AXL expression

To observe differences in functional activation of cell-types between patient groups, we performed differential expression analysis on state marker expression within each cell-type. In ICI OS-low patients, we observed strong pERK- and granzyme-B-upregulation in *i*-neutrophils (**Figure 6B**). This indicates that not only increased abundance, but also functional activation of neutrophils contributes to poor ICI outcomes, and solidifies neutrophils as the main determinant of ICI-resistance in our IMC cohort. Furthermore, OS-low patients had higher AXL expression in tumor cells, providing orthogonal evidence of the poor ICI-prognostic effect of tAXL expression at the single-cell IMC level. OS-high patients, on the other hand, showed increased tumor-cell PD-L1 expression and *i*-CD8 granzyme-B expression. As these are some of the strongest TME-predictors of ICI-response, this finding supports the ability of IMC to distinguish the most important prognostic TME features even in a relatively small cohort of patients, and that these features are present even at early disease timepoints.

In AXL Hscore-high patients, we observed stronger Ki67 staining in all T-cell subsets (**Figure 6C**). Simultaneously, we found increased expression of pERK in neutrophils and PD-L1 on MoDCs and myeloid cells; all of which had higher intratumoral densities in tAXL-positive patients. These findings suggest that tAXL-upregulation promotes an immune-suppressed TME.

In AXL IC-positive patients, AXL expression was upregulated in *i*-CD163+ M2 macrophages (**Figure 6D**), suggesting that these cells may correspond to the AXL+ immune cells measured by IHC. *i*-CD163+ macrophages also showed elevated levels of the secreted AXL-ligand Gas6, which may be a surrogate for AXL expression as Gas6-AXL is proposed to exist as a membrane dimer (34), or is indicative of autocrine-signaling. Furthermore, we found increased expression of PD-L1 on *i-*DC-LAMP+ cells, indicating their activation and maturation, while pERK was downregulated in immunosuppressive *i*-Tregs and *i*-neutrophils, altogether supporting AXL IC-score as a positive ICI-prognostic.

In summary, our IMC cohort provides mechanistic evidence that tAXL-upregulation and adverse mutations modulate the TME along an axis towards infiltrating, activated neutrophils and away from anti-tumor immune populations including CD4 and CD8 T-cells which associated with ICI survival, high PDL1 TPS, and AXL IC-positivity.

## 4 Discussion

Primary and acquired resistance to ICI therapy remains a barrier to durable clinical benefit in NSCLC patients. AXL is uniquely associated with both tumor intrinsic and immune resistance mechanisms. Surprisingly, we found differences in the predictive value of tumor- versus immune-AXL expression. Current data on the magnitude and frequency of tAXL expression in NSCLC is incomplete and inconsistent (summarized in **Supplementary Table 7**). Earlier AXL-IHC studies in NSCLC use different scoring methods and different anti-AXL antibodies, most of which are polyclonal and thus can exhibit nonspecific binding and lot-to-lot variability. We used a rigorously-validated monoclonal antibody coupled with Hscore, a more quantitative approach used previously to describe tAXL expression in IHC-stained NSCLC tissues (35). Reported tAXL-positive rates vary widely from 27%-92% (**Supplementary Table 7**). Our tAXL-positive rate of 49% places our study in the lower region of published results. Notably, NSCLC samples analyzed in former studies are mostly derived from early-stage lobectomies. In contrast, most of our patients had metastatic disease, and some biopsies were derived from distant metastases and/or pretreated with chemotherapy.

Previous studies describing tAXL in NSCLC focused mostly on adenocarcinomas; although several included squamous-cell carcinomas, no differences in tAXL expression between squamous and non-squamous tumors were reported (**Supplementary Table 7**). Our study is the first to our knowledge to report significantly lower tAXL expression in advanced NSCLC with squamous vs non-squamous histologies. Distinct cell populations of the airway and alveolar compartments are maintained by tissue resident progenitor cells which plays a crucial role in homeostasis, repair and cancer. Induced AXL signaling and tightly regulated AXL signaling has been implicated in cell-cycle re-entry of quiescent stem cells and maintenance of an undifferentiated state (5, 9, 36). Recent reports have added to the complexity of the putative cells of origin of non-squamous lung carcinomas (37, 38) and have highlighted the role of mediators of plasticity and cell faith regulators. On this background we hypothesize that the higher tAXL expression in non-squamous lung carcinomas reflects the role of AXL in restricting AT1 cell differentiation programme and mediating symmetric amplification rather than reflecting the constitutive expression of AXL in the putative stem cells of origin for the respective subtypes.

AXL regulates key processes in cancer development (9–11) and is associated with acquired resistance mechanisms to ICI-therapy by contributing to an immune-evasive TME (39). In our study, we found a strong association between PD-L1 and tAXL expression across all NSCLC subgroups, consistent with previous results suggesting that AXL-signaling upregulates PD-L1 in different malignancies (15, 16, 40). AXL-signaling has also been shown to prevent cytotoxic T-cells from binding and killing NSCLC cells (17) and is an important negative-feedback regulator of type I interferon-driven antitumor immunity (5), enabling cancers to resist ICI-mediated T-cell cytotoxicity in the long-term.

In addition to its direct effects on antitumor immunity, AXL is involved in key processes of EMT and migration, enabling metastasis (5). We found higher tAXL expression in distant metastases compared to primary lung tumors. tAXL expression was also higher in patients with brain metastases, confirming results describing higher tAXL expression in NSCLC patients with brain metastases versus those with metastases to other organs (35).

The association between tAXL expression and poor ECOG-performance status, larger tumors, and inflammation suggests that AXL-signaling elicits an aggressive cancer phenotype. This was also evident in the 1L-cohort, where tAXL expression was higher in patients receiving RT to treat aggressive local tumor growth or bone metastases. The association between tAXL expression and chemoresistance in our 2L cohort confirms prior reports (41, 42), and implies that addition of AXL-inhibitors to current 1L-chemoimmunotherapy (CIT) regimens could prevent chemoresistance even in patients lacking pretreatment tAXL expression, as our results suggest that tAXL is also induced by chemotherapy.

The consensus from previous studies is that tAXL expression is associated with more aggressive tumors and worse outcomes. It was therefore surprising to observe that in 1L-patients and WES-patients, tAXL expression was associated with improved DCR and a trend to longer PFS, although not increased OS. In contrast, in 2L-patients, there was no effect of tAXL expression on PFS or DCR, but significantly reduced survival for patients with high tAXL expression. This suggests that chemotherapy induces more malignant effects of AXL-signaling that in turn affect ICI prognosis. Adding AXL-inhibitors to 2L-ICI regimens could reverse these effects and re-sensitize ICI-resistant tumors to ICI-therapy (17, 21).

Results from our mutational analyses provide further mechanistic evidence that NSCLC cells upregulate tAXL in response to oncogenic molecular alterations, which can trigger both ICI-essential antitumor immune responses as well as EMT-mediated immune escape and an altered immune-suppressed phenotype (43).

Patients with high tAXL and PD-L1 expression were enriched in mutations associated with ICI-response and antitumor immunity. Among these, *ZNF469* mutations were found to be targeted by neoantigen-specific T-cells in a long-term responder to CIT (44), while ANKRD27 and OTOF were identified as potential tumor-specific antigens for liver and kidney cancer, respectively (45). Furthermore, *PTPRT*-mutation correlated with favorable response, higher TMB and lymphocyte infiltration in ICI-treated melanoma and NSCLC patients (7).

MUC4 functions as a tumor-suppressor in NSCLC by inducing p53 expression and attenuating Akt-signaling, and wildtype-MUC4 expression correlates with decreasing stage and improved survival (46). In our study *MUC4* mutations, which were enriched in TMB-high and AXL/PD-L1 double-positive patients, were associated with DC and longer PFS. In the context of ICI-therapy *MUC4* has been proposed as a novel tumor-associated antigen in pancreatic cancer (47), while in colorectal cancer, *MUC4*-mutation was found to be associated with high TMB and increased T-cell infiltration (48). Furthermore, a recent study detected peripheral, MUC4-neoantigen-specific CD8 T-cells in metastatic epithelial cancer patients (49). The same study also found circulating memory T-cells targeting oncogenic *KRAS* mutations in half of patients tested, while in our WES cohort, *KRAS* mutations were likewise-enriched in AXL/PDL1 double-positive patients. Thus, the unexpected association between tAXL expression and ICI-responsiveness observed in this study may be driven by oncogenic variants in genes such as *MUC4* and *KRAS* that elicit neoantigen-specific anti-tumor immune responses, prompting AXL/PD-L1-upregulation and priming tumors to respond to ICIs. Our study is the first to our knowledge to report *MUC4*-mutation as a positive-predictive biomarker for ICIs in NSCLC, although further validation studies are needed.

Conversely, we also found mutations enriched in tAXL-high patients that were associated with poor ICI outcomes and features of ICI-resistance. Mutations in *CSMD1* and *LRP1B* were enriched in tAXL-high/PD-L1-low patients and associated with shorter OS within 2L-PD-L1-low patients. Both genes are known tumor-suppressors whose inactivation is associated with EMT-related processes of invasion, migration, and poor prognosis in a variety of cancers (50, 51). *PEG3*- and *SMARCA2*-mutation also correlated with tAXL-upregulation. *PEG3*-knockdown has been shown to enhance somatic cell reprogramming (52), while SMARCA2-deficiency correlated with poor NSCLC prognosis (53). Furthermore, we found *LRP1B*-, *PEG3*-, and *SMARCA2*-mutated tumors had higher ratios of immunosuppressive neutrophils, MoDCs and Tregs and reduced CD8 T-cells within the immune compartment.

Congruently, tAXL-positive tumors were highly-enriched in activated, infiltrating neutrophils, MoDCs and myeloid cells, while simultaneously exhibiting strong upregulation of the proliferation marker Ki67 in all T-cell subsets. Single-cell RNA- and TCR-sequencing of NSCLC specimens has shown that expansion of tumor-specific T-cell clones is marked by their increased proliferation within the tumor (31). This suggests that T-cells have recognized these tAXL-positive tumors, and that AXL-signaling (in concert with EMT-enabling mutations) recruits immunosuppressive mediators to the TME to attenuate this response.

Interestingly, growing evidence suggests that both *CSMD1* and *LRP1B* mutations correlate with favorable ICI outcomes and high TMB (50, 51). Indeed, *LRP1B* (and *PEG3*) mutations were enriched in TMB-high patients in our study. However, TMB had no effect on ICI outcomes, and was conversely higher in patients with immune-cold tumors which responded poorly to ICI. Due to their increased mutational burden, these immune-cold tumors had more enriched gene mutations than any other subgroup besides high TMB itself; some of which, including the EMT-suppressor *PCDH8* (54), had independent adverse effects on ICI outcomes. Thus, we hypothesize that favorable immunogenic effects of high TMB are counteracted by positive selection of malignant clones that gain immune-evasive abilities via EMT-enabling mutations and tAXL-upregulation, which has also been shown to directly increase adaptive mutagenicity *in-vitro* and correlate with TMB in NSCLC (55). This is supported by the fact that TMB-high patients were enriched in both favorable mutations such as *MUC4* and *TP53* as well as adverse mutations such as *LRP1B* and *COL11A1*. Furthermore, TMB-high patients had increased immune-ratios of infiltrating CD4 T-cells, Tregs and B-cells; yet CD8 T-cells, the most critical anti-tumor immune subset, were markedly reduced. Overall, these findings highlight the shortcomings of TMB as a predictive biomarker for ICI-therapy and suggest that mutational panels and AXL-IHC may have superior clinical utility.

In conclusion, our study provides evidence for the involvement of AXL in both positive-predictive and negative-prognostic processes in ICI-treated NSCLC patients. On one hand, AXL is upregulated by tumor cells in response to antitumor immunity and induces PD-L1 expression, priming tumors to initially respond to ICIs. Conversely, AXL-signaling potentiates other EMT-related molecular programs that promote chemoresistance, metastasis and recruitment of inflammatory mediators which ultimately proves detrimental to ICI prognosis. The immunosuppressive effects of tAXL on the TME observed in this study combined with its apparent upregulation during chemotherapy has direct implications for current 1L-CIT regimens. Combined CIT and AXL inhibition may prolong responses by subverting AXL-mediated ICI-resistance, especially in PD-L1-low and -negative patients. Current and future trials combining AXL-inhibitors with CIT regimens are warranted to determine if NSCLC patients can benefit from such therapies.

## Data Availability

The original contributions presented in the study are included in the article/**Supplementary Material**, further inquiries can be directed to the corresponding author. Restrictions apply to the genomic sequence data: The datasets presented in this article are not readily available for patient privacy reasons. Requests to access the datasets should be directed to david.micklem@bergenbio.com and will be complied with subject to Regional Ethical Committee approval.
