# Supplementary material for: AXL expression reflects tumor-immune cell dynamics impacting outcome in non-small cell lung cancer patients treated with immune checkpoint inhibitor monotherapy
Source: Front Immunol. 2024 Aug 21;15:1444007. doi: 10.3389/fimmu.2024.1444007 (PMC11375292; doi:10.3389/fimmu.2024.1444007)
Supplement: Supplementary file 1 [file Image1.pdf]

Figure S1

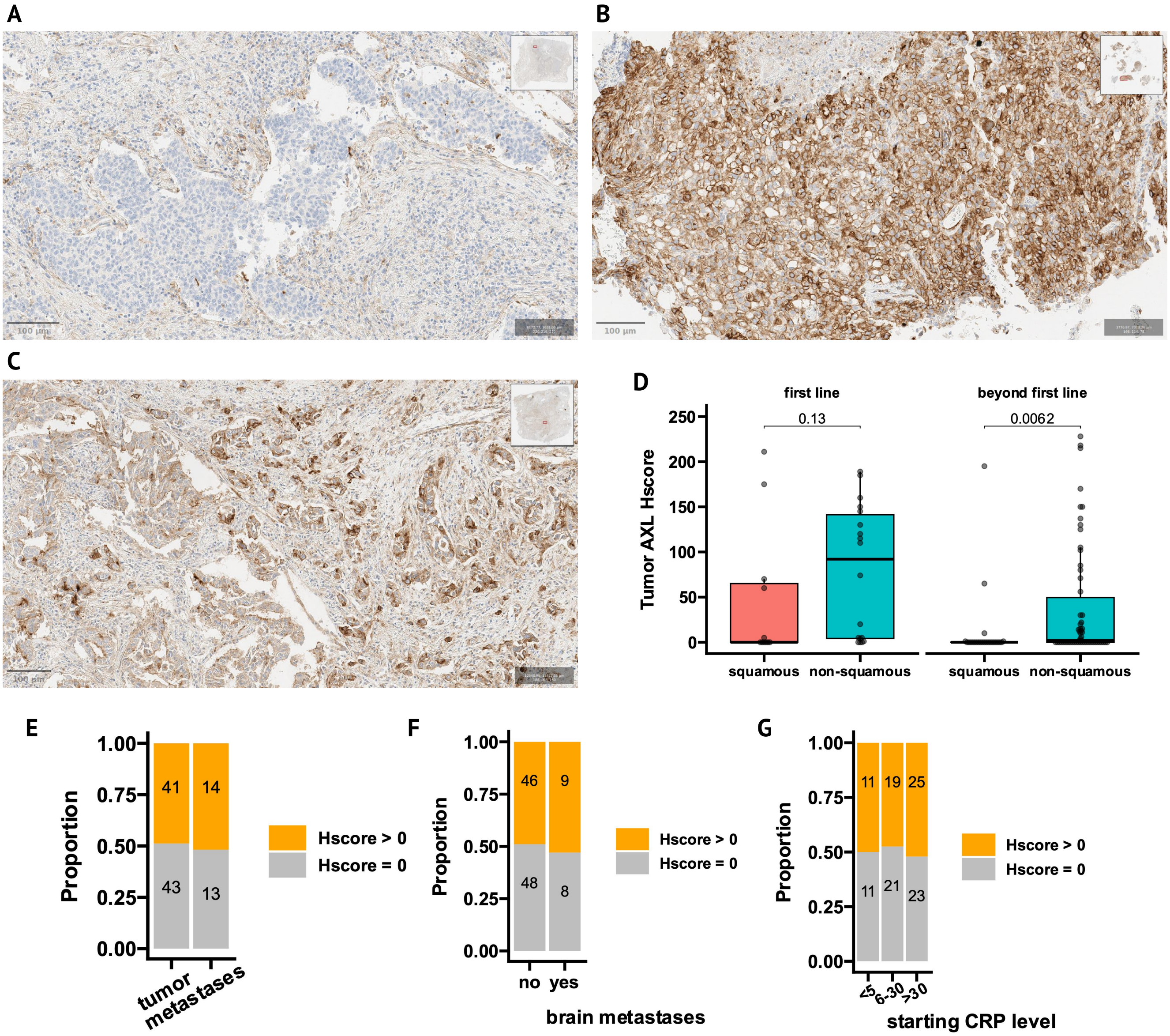

Figure S1. Tumor AXL expression in NSCLC patient biopsies.
A, Representative AXL IHC staining image of an NSCLC squamous cell carcinoma surgical resection specimen showing negative tumor-cell AXL expression (Hscore=0). Within the tumor margin, rare AXL+ infiltrating immune cells are visible (AXL IC score = 2%) while all tumor cells are negative. **B-C,** Representative AXL IHC staining images of NSCLC squamous cell carcinoma **(B)** and adenocarcinoma **(C)** surgical resection specimens showing strong-positive tumor-cell AXL expression (Hscore = 195 and 170, respectively). Across the entire tissue section shown in **C**, 5% of tumor cells were AXL-negative, 20% showed weak cytoplasmic staining, and 75% showed moderate staining, yielding an AXL Hscore of (20*1 + 75*2) = 170. Scalebar = 100µm. **D,** Boxplots of AXL Hscore in patients grouped by line of ICI therapy, stratified by histology. **E-G,** Barplots showing that the proportion of AXL-negative biopsies was roughly equal in metastatic vs primary NSCLC biopsies **(E)** in patients with or without brain metastases **(F)** and between patients stratified by starting CRP level **(G).**
