# Supplementary material for: AXL expression reflects tumor-immune cell dynamics impacting outcome in non-small cell lung cancer patients treated with immune checkpoint inhibitor monotherapy
Source: Front Immunol. 2024 Aug 21;15:1444007. doi: 10.3389/fimmu.2024.1444007 (PMC11375292; doi:10.3389/fimmu.2024.1444007)
Supplement: Supplementary file 2 [file Image2.pdf]

Figure S2

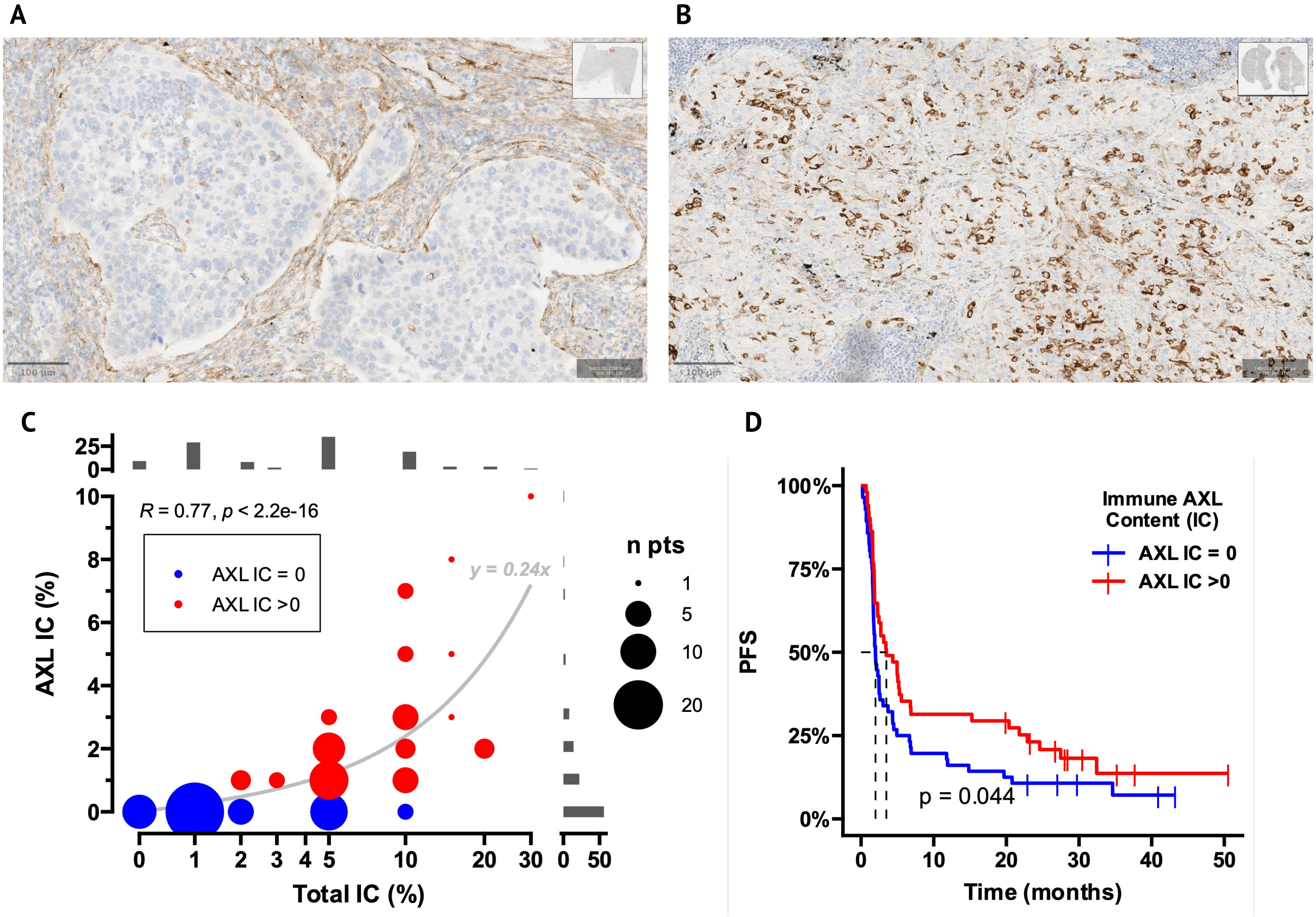

Figure S2. Immune AXL expression and ICI outcomes.

A-B, Representative AXL IHC staining images of NSCLC squamous cell carcinoma (**A**) and adenocarcinoma (**B**) surgical resection specimens showing low/negative and high levels of AXL+ immune cell infiltration within the tumor margin (AXL IC score = 0 (<1%) and 10%, respectively). Both specimens were tAXL-negative (Hscore = 0). Scalebar = 100µm. **C,** Scatter plot of AXL vs Total IC score (% AXL+ or total immune cells per tumor area). Due to many patients having low total and AXL IC scores, total IC was plotted on a log scale, and histograms along edges of plot show the number of patients with a given AXL or total IC score. Point size represents the number of patients (n pts) with a specific combination of total and AXL IC score. Grey line represents the best-fit linear regression line with x-intercept fixed at zero. **D,** Progression-free survival (PFS) of patients stratified by presence of measurable AXL+ tumor-infiltrating immune immune cells by IHC (AXL IC ≥1%). R and p-values in scatter and survival plots from spearman correlation and log-rank test, respectively.
