# Supplementary material for: AXL expression reflects tumor-immune cell dynamics impacting outcome in non-small cell lung cancer patients treated with immune checkpoint inhibitor monotherapy
Source: Front Immunol. 2024 Aug 21;15:1444007. doi: 10.3389/fimmu.2024.1444007 (PMC11375292; doi:10.3389/fimmu.2024.1444007)
Supplement: Supplementary file 3 [file Image3.pdf]

Figure S3

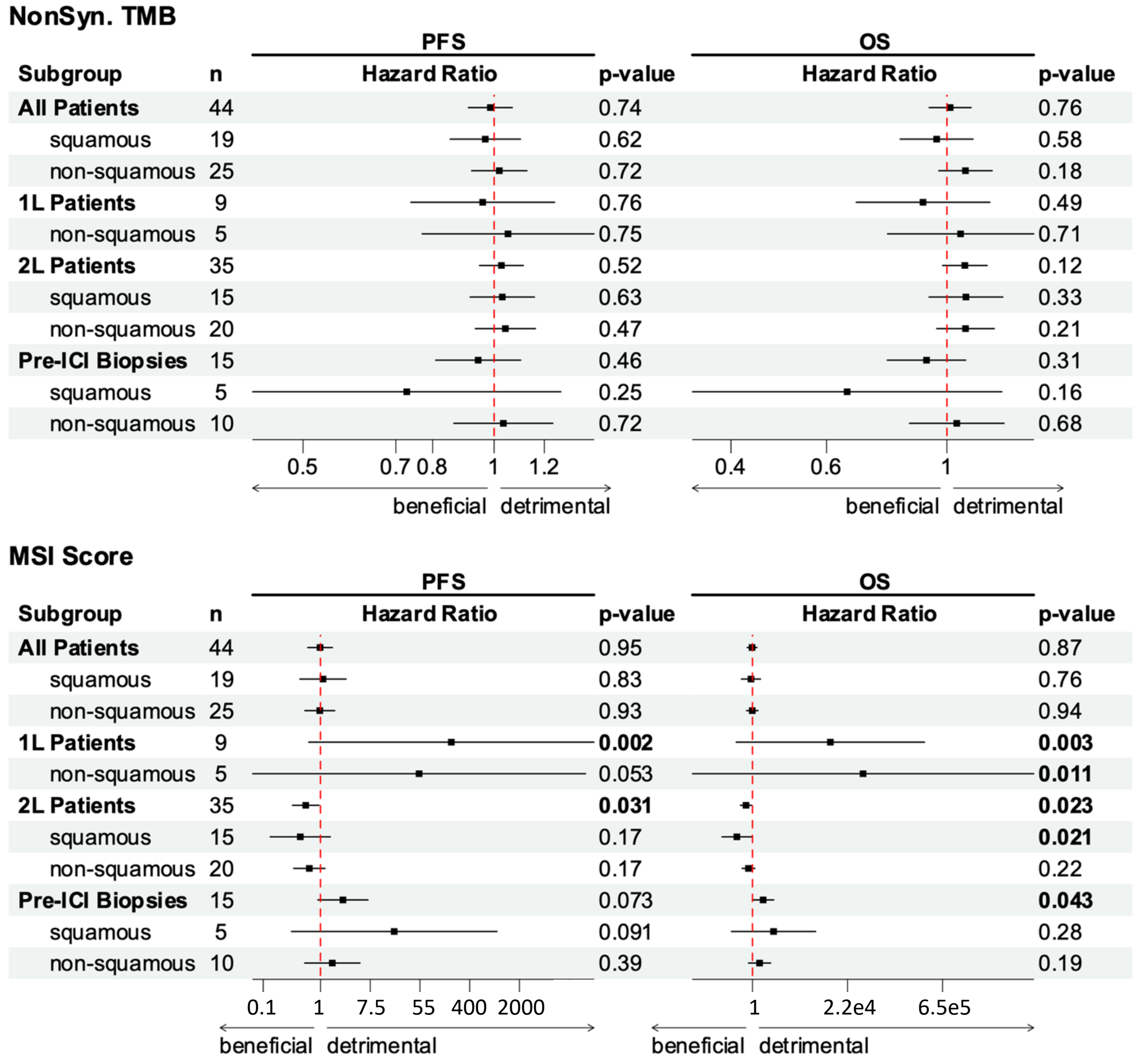

Figure S3. Association of TMB and MSI score with ICI outcomes.

Hazard Ratio plots showing the effect of non-synonymous tumor mutational burden (TMB, mutations per megabase, top) and MSI score (percentage *de novo* microsatellite sites, bottom) as continuous variables on PFS and OS for all WES patients and subgroups stratified by line of ICI therapy and histology. Biopsies from 2L ICI patients taken after 1L systemic therapy were grouped with 1L ICI patient biopsies to represent biopsies taken directly before ICI treatment (Pre-ICI biopsies). Hazard ratios and p-values from univariate cox regression. Subanalyses of 1L squamous patients failed due to too few patients (n=4).
