# Supplementary material for: AXL expression reflects tumor-immune cell dynamics impacting outcome in non-small cell lung cancer patients treated with immune checkpoint inhibitor monotherapy
Source: Front Immunol. 2024 Aug 21;15:1444007. doi: 10.3389/fimmu.2024.1444007 (PMC11375292; doi:10.3389/fimmu.2024.1444007)
Supplement: Supplementary file 4 [file Image4.pdf]

## Figure S4

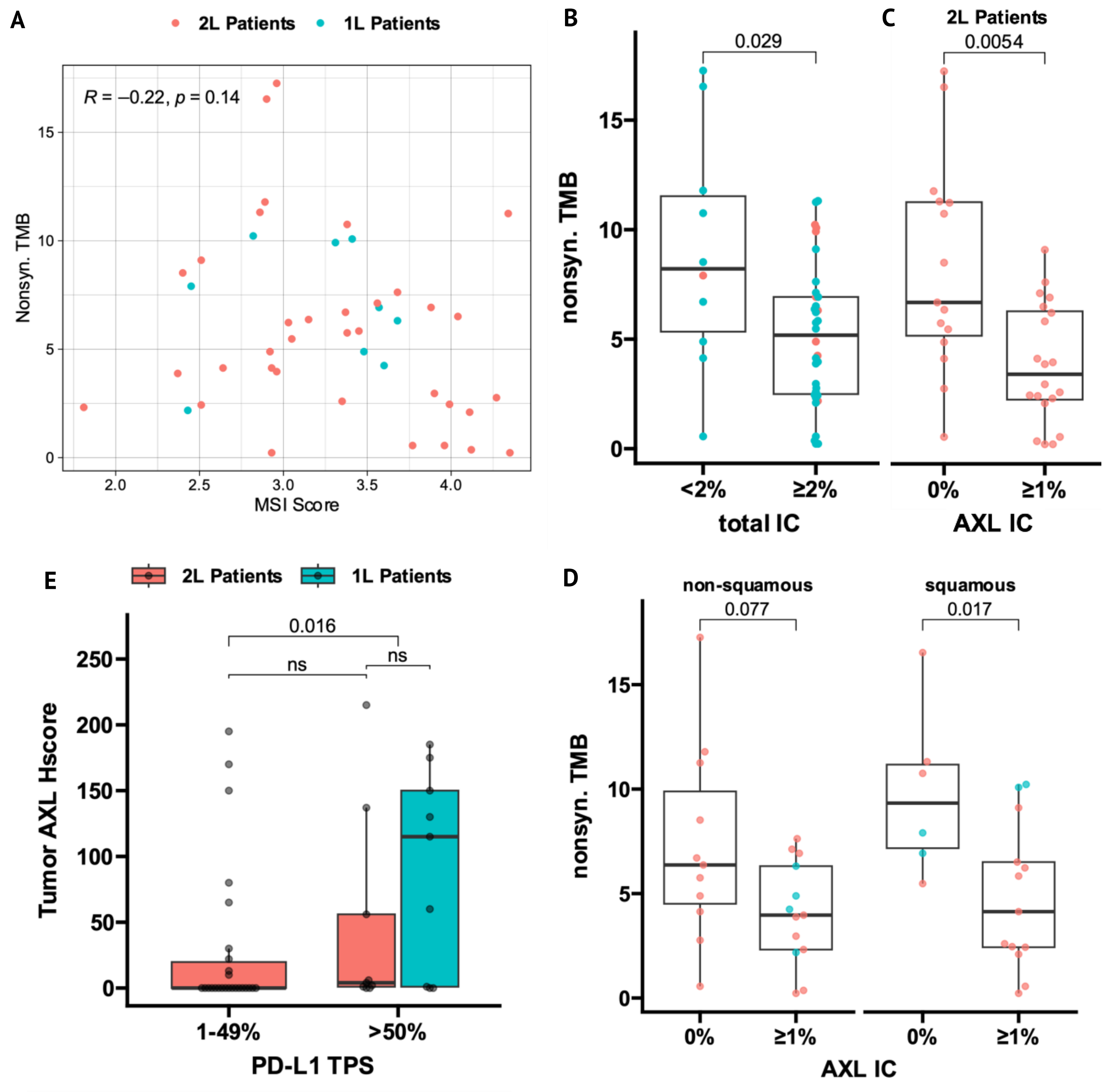


**Figure S4. Immune AXL expression and ICI outcomes.**

**A,** Scatter plot of MSI score (percentage *de novo* microsatellite sites) versus non-synonymous tumor mutational burden (TMB, mutations per megabase) per patient, showing no correlation. **B-C,** Boxplots showing TMB levels are higher in patients with few total infiltrating immune cells (total IC<2%) **(B)**, and in patients with no detectable AXL+ infiltrating immune cells (AXL IC = 0) within 2L **(C)** and histology **(D)** subgroups. **E,** boxplot showing AXL Hscores are higher in WES patients with high vs low PD-L1 TPS. R and p-values in scatter and box plots from Spearman correlation and Mann-Whitney U-test, respectively. ns: not significant (p>0.1)
