# Supplementary material for: AXL expression reflects tumor-immune cell dynamics impacting outcome in non-small cell lung cancer patients treated with immune checkpoint inhibitor monotherapy
Source: Front Immunol. 2024 Aug 21;15:1444007. doi: 10.3389/fimmu.2024.1444007 (PMC11375292; doi:10.3389/fimmu.2024.1444007)
Supplement: Supplementary file 5 [file Image5.pdf]

# Figure S5

**A** **Single-gene mutation enrichments:**

squamous (n = 19) v/s non-squamous (n = 25)

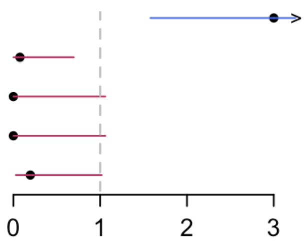

| | squamous | non-squamous | OR | P-value |
|---|---|---|---|---|
| *KRAS* | 0 | 8 | Inf | ** |
| *KMT2D* | 7 | 1 | 0.076 | * |
| *FMN2* | 4 | 0 | 0 | * |
| *PIK3CA* | 4 | 0 | 0 | * |
| *USH2A* | 8 | 3 | 0.195 | * |

*Odds ratio with 95% CI*
*(1 = no effect, < 1 squamous has more mutants)*

**Pathway mutation enrichments:**

squamous (n = 19) v/s non-squamous (n = 25)

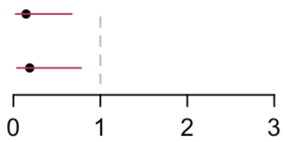

| | squamous | non-squamous | OR | P-value |
|---|---|---|---|---|
| *NRF2* | 11 | 4 | 0.146 | ** |
| *PI3K* | 13 | 7 | 0.188 | * |

**B** **pAXLIC≥1 (n = 26) v/s pAXLIC <1 (n = 17)**

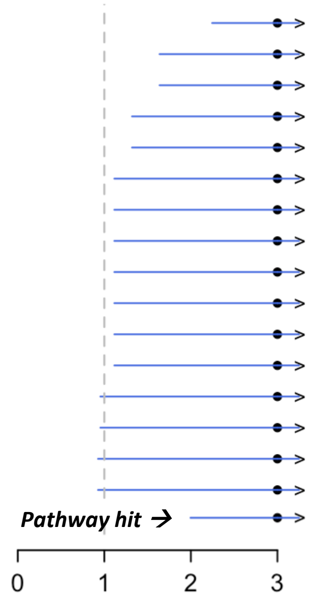


| | pAXLIC≥1 | pAXLIC <1 | OR | P-value |
|---|---|---|---|---|
| *PCDH8* | 0 | 6 | Inf | ** |
| *MDN1* | 0 | 5 | Inf | ** |
| *TENM3* | 0 | 5 | Inf | ** |
| *ANK2* | 1 | 6 | 12.792 | * |
| *FBN2* | 1 | 6 | 12.792 | * |
| *CD163L1* | 0 | 4 | Inf | * |
| *FMN2* | 0 | 4 | Inf | * |
| *GREB1* | 0 | 4 | Inf | * |
| *KRT76* | 0 | 4 | Inf | * |
| *LAMA1* | 0 | 4 | Inf | * |
| *NLRP14* | 0 | 4 | Inf | * |
| *SH3TC2* | 0 | 4 | Inf | * |
| *OTOG* | 1 | 5 | 9.848 | * |
| *PCDHA9* | 1 | 5 | 9.848 | * |
| *CSMD1* | 2 | 6 | 6.239 | * |
| *PAPPA2* | 2 | 6 | 6.239 | * |
| NOTCH | 8 | 14 | 9.85 | ** |

**C** **Disease control yes (n = 19) vs no (n = 25)**

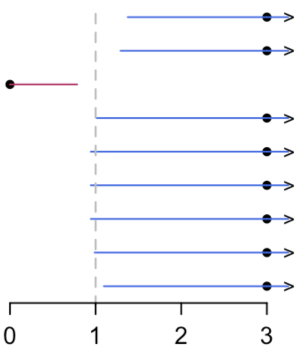

| | no | yes | OR | P-value |
|---|---|---|---|---|
| *ZNF469* | 0 | 5 | Inf | * |
| *PKHD1L1* | 2 | 8 | 7.937 | * |
| *FAT1* | 7 | 0 | 0 | * |
| *PCDH15* | 2 | 7 | 6.408 | * |
| *CSMD2* | 0 | 4 | Inf | * |
| *OTOF* | 0 | 4 | Inf | * |
| *PLXNB1* | 0 | 4 | Inf | * |
| *MUC4* | 5 | 10 | 4.281 | * |
| *COL6A3* | 1 | 6 | 10.498 | * |

**D** **AXL Hscore pos vs neg (0 vs >0)**

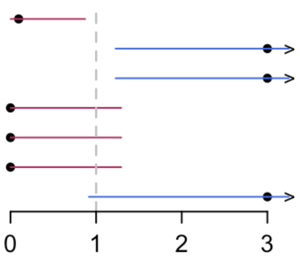

| | low | high | OR | P-value |
|---|---|---|---|---|
| *KMT2D* | 7 | 1 | 0.096 | * |
| *MACF1* | 0 | 6 | Inf | * |
| *NF1* | 0 | 6 | Inf | * |
| *DCAF8L2* | 4 | 0 | 0 | * |
| *GRM1* | 4 | 0 | 0 | * |
| *TDRD15* | 4 | 0 | 0 | * |
| *DMD* | 0 | 5 | Inf | * |

## Figure S5, continued

**E** AXL Hscore low vs high (<50 vs >50)

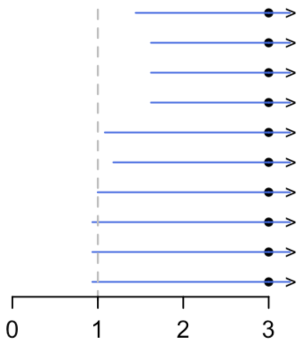


| Gene | low | high | OR | P-value |
|---|---|---|---|---|
| *MUC4* | 6 | 9 | 6.813 | ** |
| *ANKRD27* | 0 | 4 | Inf | ** |
| *MYH4* | 0 | 4 | Inf | ** |
| *TRIM63* | 0 | 4 | Inf | ** |
| *CSMD1* | 3 | 6 | 6.399 | * |
| *LRP1B* | 4 | 7 | 6.167 | * |
| *SMARCA2* | 2 | 5 | 7.348 | * |
| *PEG3* | 1 | 4 | 10.868 | * |
| *PTPRT* | 1 | 4 | 10.868 | * |
| *ZNF469* | 1 | 4 | 10.868 | * |

**F** low (n = 26) v/s high (n = 18)

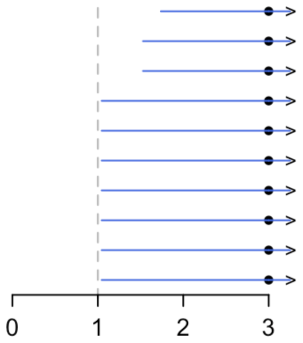


| PDL1 TPS | low | high | OR | P-value |
|---|---|---|---|---|
| *MUC4* | 4 | 11 | 8.141 | ** |
| *CDH18* | 0 | 5 | Inf | ** |
| *PTPRT* | 0 | 5 | Inf | ** |
| *ADCY10* | 0 | 4 | Inf | * |
| *ANKRD27* | 0 | 4 | Inf | * |
| *JMJD1C* | 0 | 4 | Inf | * |
| *NCKAP5* | 0 | 4 | Inf | * |
| *OTOF* | 0 | 4 | Inf | * |
| *SRRM3* | 0 | 4 | Inf | * |
| *ZNF112* | 0 | 4 | Inf | * |

**G** yes (n = 16) v/s no (n = 28)

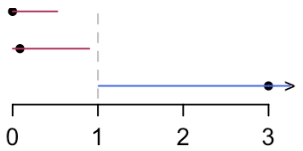


| Bone Metastases | yes | no | OR | P-value |
|---|---|---|---|---|
| *MRGPRX3* | 5 | 0 | 0 | ** |
| *PCDHA9* | 5 | 1 | 0.087 | * |
| *TTN* | 3 | 15 | 4.819 | * |

**H** yes (n = 10) v/s no (n = 34)

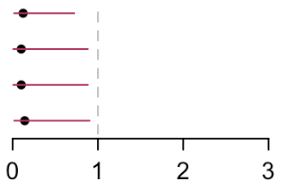


| Liver Metastases | yes | no | OR | P-value |
|---|---|---|---|---|
| *SYNE1* | 6 | 5 | 0.123 | ** |
| *NFE2L2* | 4 | 2 | 0.101 | * |
| *PCDHA9* | 4 | 2 | 0.101 | * |
| *FLG* | 5 | 4 | 0.142 | * |

**I** yes (n = 20) v/s no (n = 24)

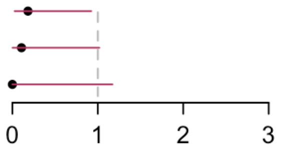


| Multiorgan Mets | yes | no | OR | P-value |
|---|---|---|---|---|
| *CSMD3* | 9 | 3 | 0.182 | * |
| *FLNC* | 6 | 1 | 0.107 | * |
| *KRT76* | 4 | 0 | 0 | * |

**J** ECOG 2-3 (n = 13) v/s ECOG 0-1 (n = 31)

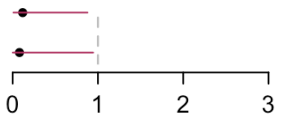


| Gene | ECOG 2-3 | ECOG 0-1 | OR | P-value |
|---|---|---|---|---|
| *FAT1* | 5 | 2 | 0.118 | * |
| *TENM3* | 4 | 1 | 0.081 | * |

**K** low (n = 22) v/s high (n = 22)

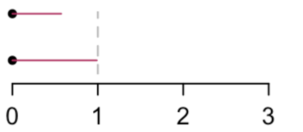


| MSI score (>median) | low | high | OR | P-value |
|---|---|---|---|---|
| *FBN3* | 7 | 0 | 0 | ** |
| *CUBN* | 5 | 0 | 0 | * |

## Figure S5, continued

**L** low (n = 22) v/s high (n = 22)

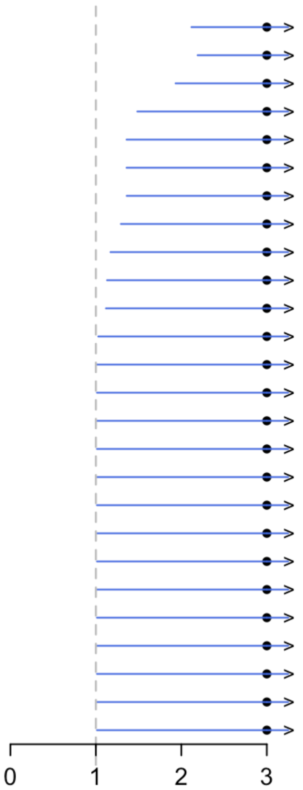


| TMB (>median) | low | high | OR | P-value |
|---|---|---|---|---|
| *MUC16* | 3 | 14 | 10.386 | ** |
| *FBN2* | 0 | 8 | Inf | ** |
| *LRP1B* | 1 | 10 | 16.442 | ** |
| *MUC4* | 3 | 12 | 7.225 | ** |
| *CSMD3* | 2 | 10 | 7.93 | * |
| *MUC19* | 2 | 10 | 7.93 | * |
| *CPS1* | 0 | 6 | Inf | * |
| *COL11A1* | 1 | 8 | 11.397 | * |
| *TP53* | 10 | 18 | 5.18 | * |
| *TTN* | 5 | 13 | 4.721 | * |
| *USH2A* | 2 | 9 | 6.623 | * |
| *NAV3* | 1 | 7 | 9.352 | * |
| *AHNAK* | 0 | 5 | Inf | * |
| *DLG2* | 0 | 5 | Inf | * |
| *DST* | 0 | 5 | Inf | * |
| *GREB1* | 0 | 5 | Inf | * |
| *GRIN2B* | 0 | 5 | Inf | * |
| *INPP5D* | 0 | 5 | Inf | * |
| *KCND2* | 0 | 5 | Inf | * |
| *MICAL3* | 0 | 5 | Inf | * |
| *MYO16* | 0 | 5 | Inf | * |
| *PCDHGA6* | 0 | 5 | Inf | * |
| *PEG3* | 0 | 5 | Inf | * |
| *PLCH1* | 0 | 5 | Inf | * |
| *SCN10A* | 0 | 5 | Inf | * |
| *TNR* | 0 | 5 | Inf | * |

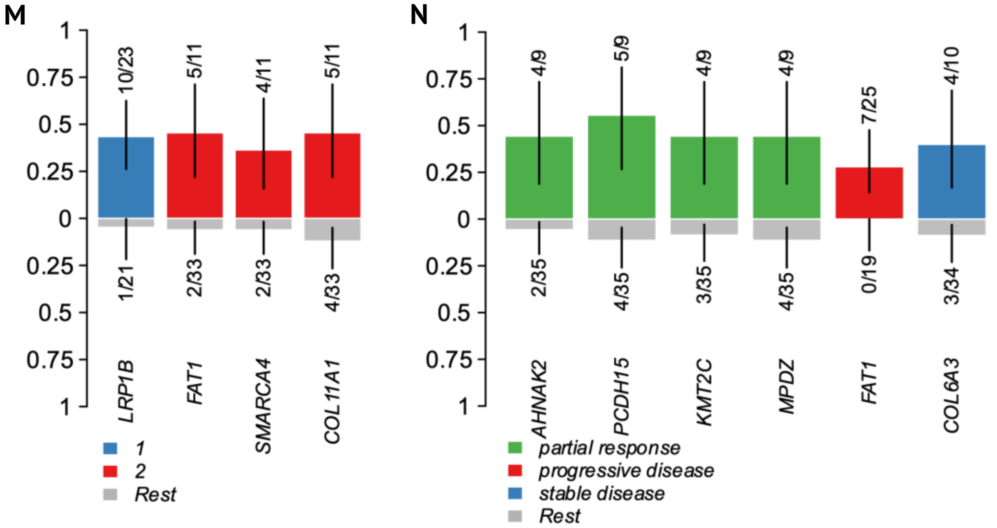


**Figure S5. Gene mutation enrichment in patient and biomarker groups**

**A-L,** Odds-ratio (OR) plots of gene mutations occurring more frequently in squamous vs non-squamous biopsies **(A)**, biopsies with vs without measurable AXL+ infiltrating immune cells (AXL IC >0 vs 0) **(B)**, patients with vs without disease control on ICI **(C)**, AXL Hscore-positive (Hscore>0) vs low (Hscore=0) **(D)**, AXL Hscore-high (>50) vs -low (<50) biopsies **(E)**, PDL1 TPS high (≥50%) vs low (1-49%) **(F)**, patients with vs without bone metastases **(G)**, liver metastases **(H)**, or metastases in multiple organ systems (multiorgan mets) at ICI start **(I)**, high (2-3) vs low (0-1) ECOG performance score **(J)**, high (above median) vs low MSI score **(K)**, and TMB **(L)**. For **A** and **B**, enrichment of mutations in known cancer signaling pathways are also shown below. Lines represent 95% confidence interval of OR. **M-N,** mutations significantly ($X^2$ $p < 0.05$) enriched in a single group for variables which contain more than two groups including ECOG score **(M)** and best response on ICI **(N)**. (*$X^2$ $p < 0.05$; **$X^2$ $p < 0.01$)
