# Supplementary material for: AXL expression reflects tumor-immune cell dynamics impacting outcome in non-small cell lung cancer patients treated with immune checkpoint inhibitor monotherapy
Source: Front Immunol. 2024 Aug 21;15:1444007. doi: 10.3389/fimmu.2024.1444007 (PMC11375292; doi:10.3389/fimmu.2024.1444007)
Supplement: Supplementary file 6 [file Image6.pdf]

Figure S6

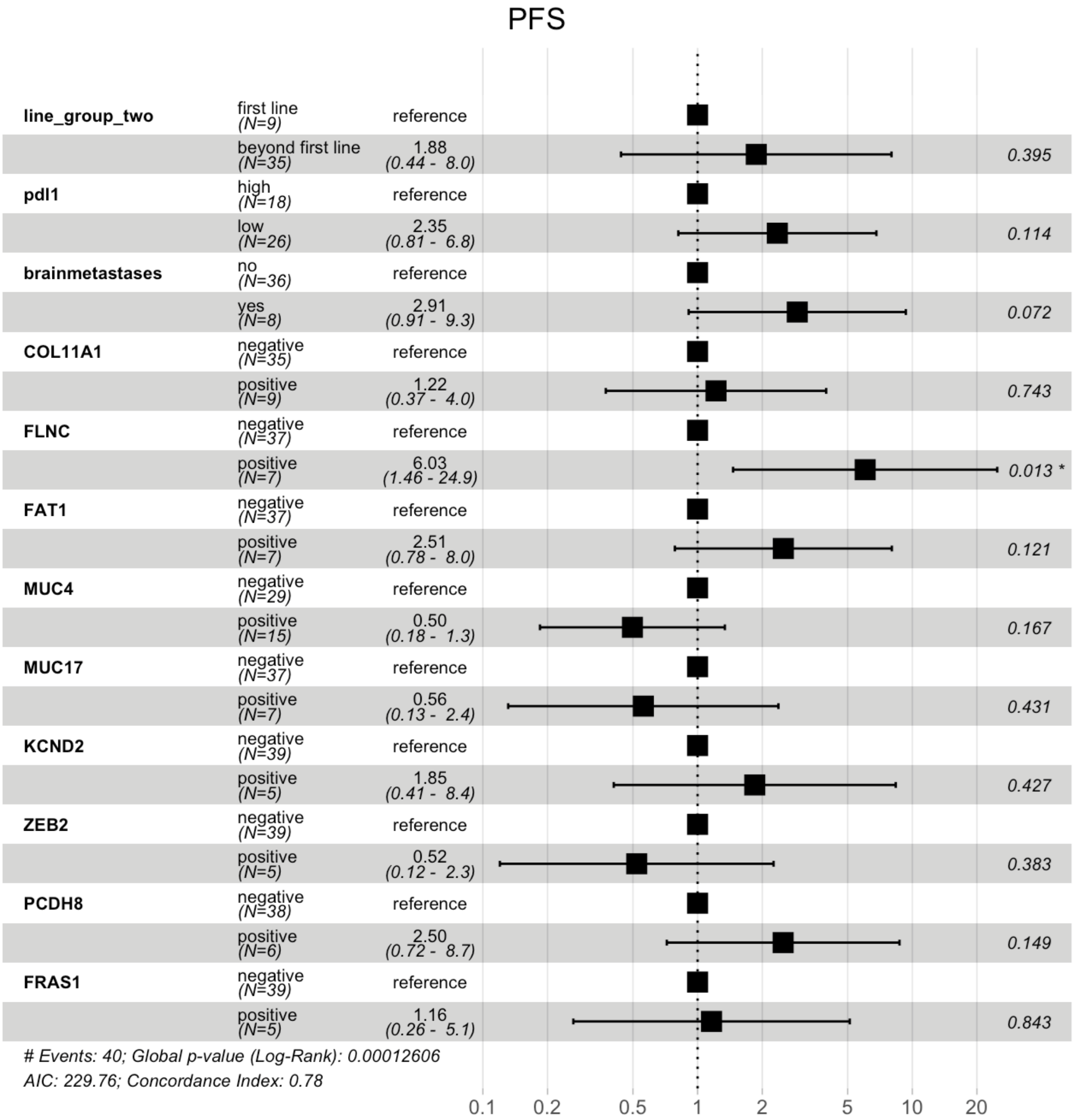

PFS
line_group_two
first line (N=9) reference
beyond first line (N=35) 1.88 (0.44 - 8.0) 0.395
pdl1
high (N=18) reference
low (N=26) 2.35 (0.81 - 6.8) 0.114
brainmetastases
no (N=36) reference
yes (N=8) 2.91 (0.91 - 9.3) 0.072
COL11A1
negative (N=35) reference
positive (N=9) 1.22 (0.37 - 4.0) 0.743
FLNC
negative (N=37) reference
positive (N=7) 6.03 (1.46 - 24.9) 0.013 *
FAT1
negative (N=37) reference
positive (N=7) 2.51 (0.78 - 8.0) 0.121
MUC4
negative (N=29) reference
positive (N=15) 0.50 (0.18 - 1.3) 0.167
MUC17
negative (N=37) reference
positive (N=7) 0.56 (0.13 - 2.4) 0.431
KCND2
negative (N=39) reference
positive (N=5) 1.85 (0.41 - 8.4) 0.427
ZEB2
negative (N=39) reference
positive (N=5) 0.52 (0.12 - 2.3) 0.383
PCDH8
negative (N=38) reference
positive (N=6) 2.50 (0.72 - 8.7) 0.149
FRAS1
negative (N=39) reference
positive (N=5) 1.16 (0.26 - 5.1) 0.843
Events: 40; Global p-value (Log-Rank): 0.00012606
AIC: 229.76; Concordance Index: 0.78
0.1
0.2
0.5
1
2
5
10
20

Figure S6, continued

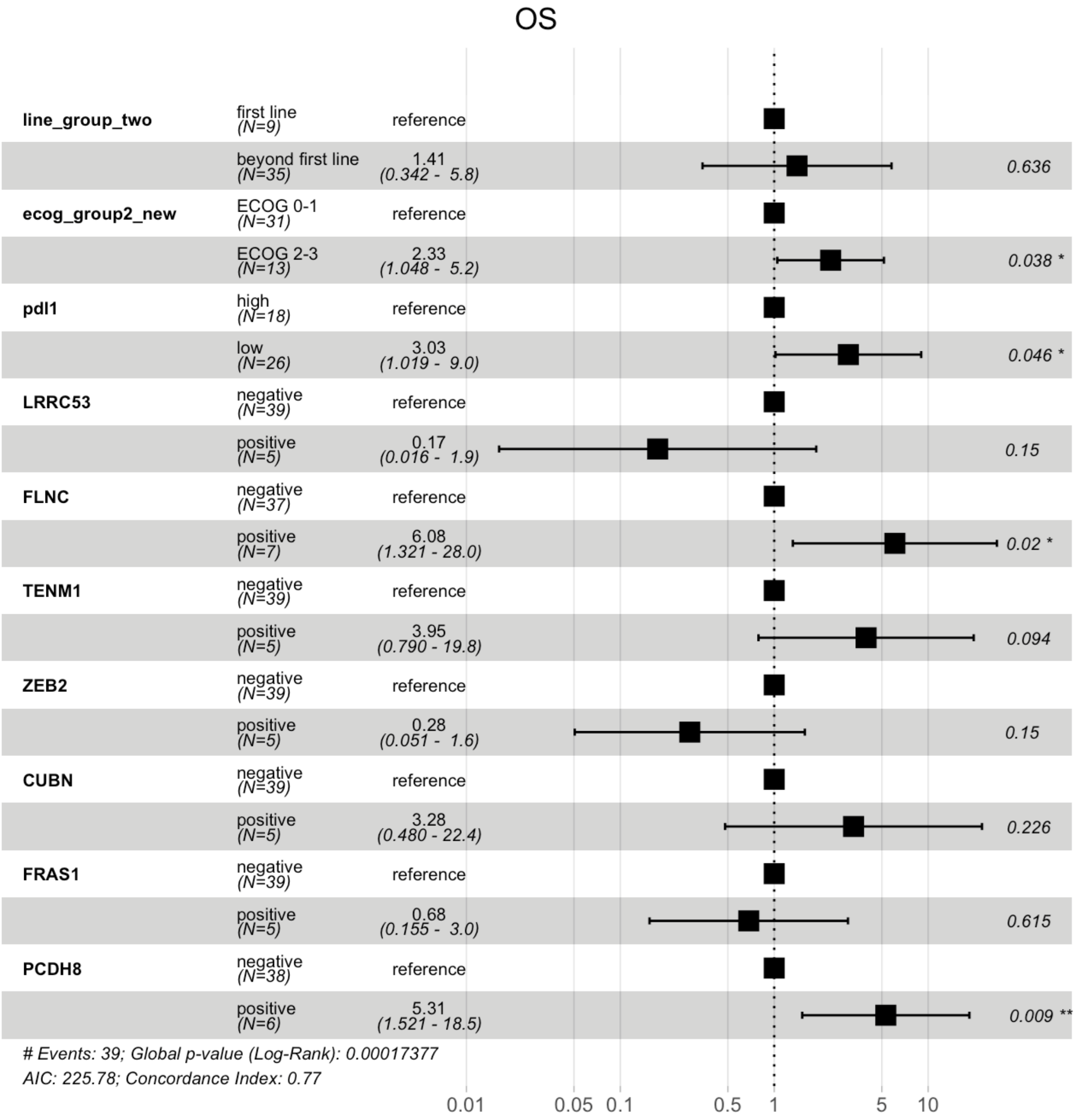

Figure S6. Multivariate survival analysis of gene mutations
All genes significantly associated with PFS or OS ($p<0.05$) in univariate analyses were tested together in corresponding multivariate cox proportional hazards models adjusted for significant clinical predictors within the WES cohort comprising line of therapy (1L vs 2L), PD-L1 TPS high vs low, presence of brain metastases (PFS only), and ECOG group (0-1 vs 2-3, OS only).
