# Supplementary figures and images for: AXL expression reflects tumor-immune cell dynamics impacting outcome in non-small cell lung cancer patients treated with immune checkpoint inhibitor monotherapy
Source: Front Immunol. 2024 Aug 21;15:1444007. doi: 10.3389/fimmu.2024.1444007 (PMC11375292; doi:10.3389/fimmu.2024.1444007)

Figure S7

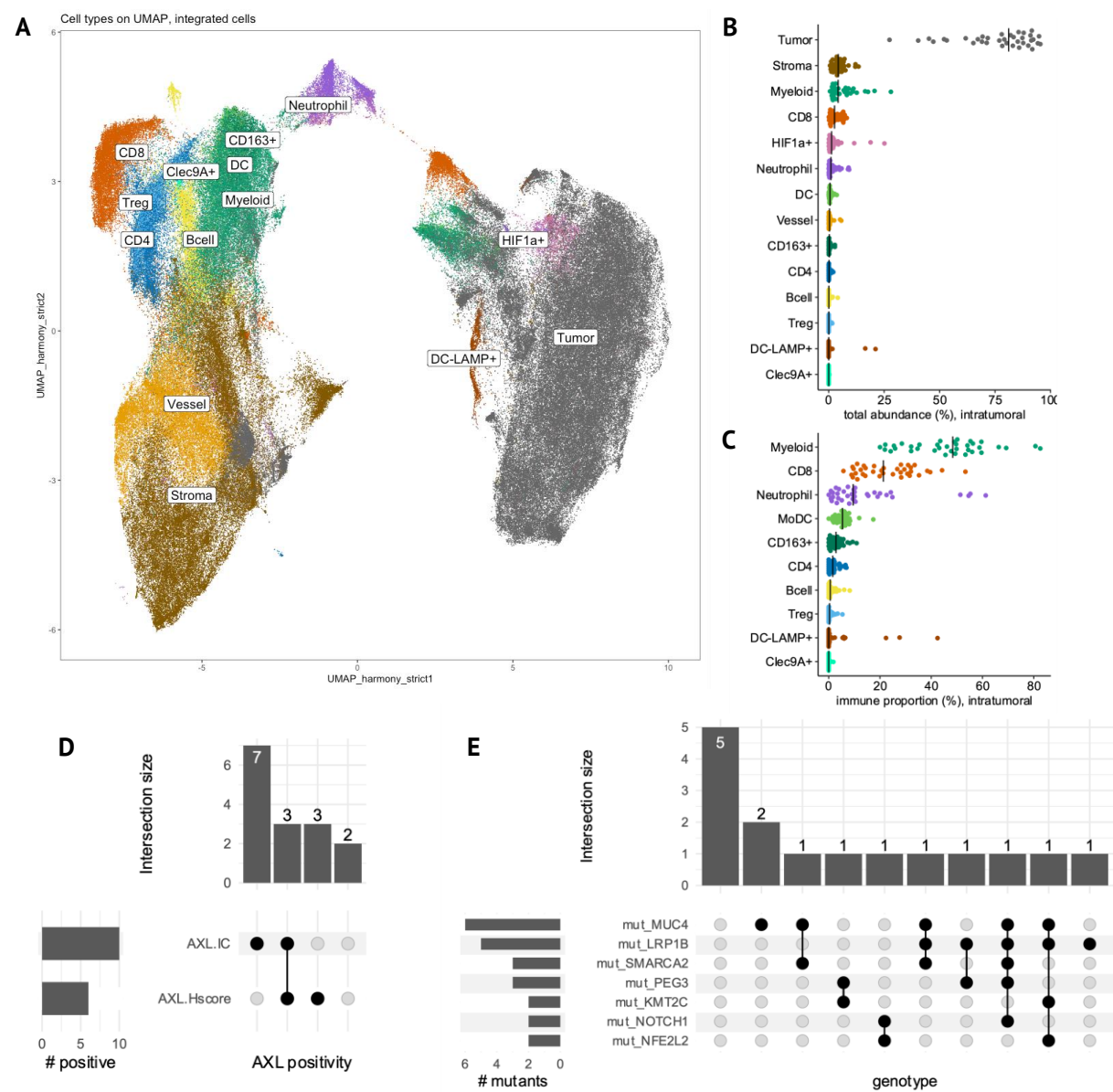

Supplement: Supplementary file 7 [file Image7.pdf]
