# Supplementary material for: AXL expression reflects tumor-immune cell dynamics impacting outcome in non-small cell lung cancer patients treated with immune checkpoint inhibitor monotherapy
Source: Front Immunol. 2024 Aug 21;15:1444007. doi: 10.3389/fimmu.2024.1444007 (PMC11375292; doi:10.3389/fimmu.2024.1444007)
Supplement: Supplementary file 14 [file Table7.docx]

Table S7. Tumor AXL expression by immunohistochemistry in lung cancer tissues – literature review

| Year/ author | Antibody (Supplier) | Histology | Stage | Biopsy type | Scoring system | Main results |
| --- | --- | --- | --- | --- | --- | --- |
| 2005,  Shieh YS et al. [1] | Rabbit anti–human Axl antibody  (Santa Cruz Biotechnology, Santa Cruz, CA) | Adenocarcinoma, n=58 | I, n=20  II-IV, n=38  No differentiation between stage II-IV. | Not described | Semiquantitative scoring.  Classification into AXL-positive if >25% of cancer cells were stained and negative if <25% of cancer cells were stained. | Axl positive rate: 48%  Axl expression associated with lymph node involvement and higher clinical stage (stage I vs II-IV): No association between AXL expression and gender, tumor size, and differentiation. |
| 2012,  Lund-Iversen M et al. [2] | Anti-AXL monoclonal antibody  (R&D Systems Inc., Minneapolis, MN, USA) | NSCLC, n=127 | I-III, n=127 | Surgical resection specimens | Ordinal scale, range 0-2.  Classification into  negative (0), intermediate (1), and strong (2) positive. | Axl positive rate: 85%  Increased risk for relapse in the AXL positive group. No significant correlation between AXL expression and age, histology, or smoking history. |
| 2013,  Ishikawa M et al. [3] | Rabbit anti-Axl polyclonal antibody  (Santa Cruz Biotechnology, Santa Cruz, CA) | Adenocarcinoma, n=88  including EGFR mutated adenocarcinomas (n=39) | I, n=54  II, n=8  III, n=26 | Surgical resection specimens | Semiquantitative scoring, range 0-12.  The score was obtained by multiplication of staining intensity (grade 0-3) and percentage of positive cancer cells (grade 0-4).  AXL high defined as score >7 | No AXL positive rate mentioned.  AXL high rate: 33%  Axl high was associated with less differentiated tumors,  higher p-stage (I vs II vs III), larger tumor-size (≤5 vs >5cm), lymph node metastases (n- vs n+), higher serum CEA and SLX levels and poor OS. |
| 2014,  Iida et al. [4] | Anti-phospho-monoclonal AXL antibody  (R&D Systems Inc., Minneapolis, MN, USA) | Adenocarcinoma, n=112 | I, n=79  II, n=12  III, n=21 | Surgical resection specimens | Ordinal scale, range 0-2.  Phospho-Axl immunoreactivity was classified into negative (0), weakly-positive (1), and markedly positive (2). | Phospho-AXL positive rate: 60%.  Markedly positive rate: 21%  Association between phospho-AXL positivity and poor OS and trend to higher T stage (p= 0.08).  No association between phospho-AXL positivity and age, gender, stage, and KI-67. |
| 2013,  Linger RM et al. [5] | Goat polyclonal anti-Axl antibody, AF154  (R&D Systems Inc., Minneapolis, MN, USA) | N total= 88  Squamous cell carcinoma, n=37,  Adenocarcinoma, n= 39, Large-cell undifferentiated carcinoma, n=7, Bronchoalveolær carcinoma, n=5 | I, n=43  II, n=12  III, n=23  IV, n=8 | Not described | Semiquantitative scoring, range 0-400.  H-score was calculated by multiplying the percentage of positive tumor cells (0% to 100%) by the dominant staining intensity (0 to 4). | Axl positive rate: 93%  H-score 0-4: 6%.  H-score 5-100: 21%.  Hscore 101-200: 32%  Hscore 201-300: 34%  Hscore 301-400: 6%  Axl expression was not associated with overall survival, stage, or histology. |
| 2015,  Qu XH et.al [6] | Rabbit polyclonal anti‑human AXL antibody  (Cat no. ab72069, Abcam, Cambridge, UK) | N total= 134  Adenocarcinoma, n=67,  Squamous cell carcinoma, n=67 | I, n=23  II-IV, n=111 | Surgical resection specimens | Semiquantitative score, range 1-9.  The score was calculated by multiplying the  proportion of AXL positive cancer cells (grade 1-3) with the staining intensity (grade 1-3).  The final scores were classified into negative (score 1-2) and positive (score 3‑9) groups. | AXL positive rate: 54%  AXL positivity was associated with less differentiation,  higher stage (I vs II/III/IV) and poor OS.  AXL positivity was not associated with gender, age, histological type, lymph node metastasis and tumor size. |
| 2016,  Sato K et al. [7] | Goat polyclonal anti-Axl antibody, AF154  (R&D Systems Inc., MN, USA) | Adenocarcinomas, n= 161 | I, n=111  II, n=25  III, n=23  IV, n= 2 | Surgical resection specimens | Ordinal scale, range 0-3.  Staining intensities graded as 0 (no  staining), 1 (weak), 2 (moderate), or 3 (strong). | AXL positive rate: 65%  Strong AXL expression was associated with poor overall survival and downregulation of E-cadherin and CD44.  No association between AXL expression and gender, age, CEA level, stage, smoking status, differentiation/grade. |
| 2017,  Seike M et al.[8] | Goat polyclonal anti-Axl antibody, AF154  (R&D Systems Inc., MN, USA) | Adenocarcinoma, n=113, including EGFR mutated adenocarcinomas (n=31) | I, n=64  II-III, n=49 | Surgical resection specimens | Semiquantitative score, range 0-12.  H score calculated by multiplying the staining intensity (grade 0-3) by the  percentage of positive cancer cells (0-4; <10%, 10–25%, 25–50%, 50–75%, ≥75% positive cells).  AXL positivity  was defined as H-score ≥1. | AXL positive rate: 38%  AXL positivity associated with poor overall survival when compared to AXL negative patients.  No association between AXL expression and age, sex, smoking, T-, N- stadium, grade, post-operative recurrence, and EGFR status. |
| 2017,  Wu X et al. [9] | Rabbit antihuman AXL monoclonal antibody  (Cell Signaling Technology, #8661) | N total= 98,  Adenocarcinoma, n= 88  Non-adenocarcinoma, n=10 | I-III, n=7  IV, n=91  Patients with brain metastases (n=66) | Tumor samples from:  -lung, n=51  -brain, n=35  -other distant metastases than the brain: n=10 | Semiquantitative score, range 0-300.  H-score was obtained by multiplying the percentage of positive cancer cells (0–100%) with staining intensity  (0 -3).  AXL expression was classified as high  when the H-score was above the median cut-off score of 0.4. | AXL positive rate: 50%  AXL positivity associated with worse OS.  AXL expression was not associated with gender, age, smoking history, histology, T stage, N stage, CEA, or LDH levels. |
| 2017,  Choi et al. [10] | anti-AXL antibody  (Santa Cruz Biotechnology,  rabbit? -not mentioned) | N total=126  Adenocarcinoma, n=53  Squamous cell carcinoma, n=73 | II, n=126 | Surgical resection specimens | Semiquantitative score, range 0-6.    The percentage of positively stained tumor cells (range 0-3) and the staining intensity (range 0 to 3) were measured.  Both scores were added, and tumors were classified into negative tumors (score 0-3) and positive tumors (score 4-6). | AXL positive rate: 27%  Disease free survival was significantly shorter in the AXL-positive group compared to the AXL negative group. |
| 2018,  Zhao et al. [11] | Not mentioned | N total= 81  Adenocarcinoma, n=39  Squamous cell carcinoma, n=42 | I, n=35  II, n= 11  III, n=35 | Surgical resection specimens | Semiquantitative score, range 0-6.  Staining intensity (0-3) and proportion of stained cells (0-3; ≤ 10%, 11%-30%, 31%-50%, > 50%) were added.  AXL high was defined as a score ≥3. | No AXL positive rate mentioned.  AXL high rate: 44%  AXL high was associated with poor differentiation, higher T stage, higher pTNM stage and poor overall survival.  AXL expression was not associated with patients age, gender, and histological type. |

References Table S7:

1. Shieh, Y.S., C.Y. Lai, Y.R. Kao, S.G. Shiah, Y.W. Chu, H.S. Lee, and C.W. Wu, Expression of axl in lung adenocarcinoma and correlation with tumor progression. *Neoplasia*, 2005. 7(12): p. 1058-64.doi: 10.1593/neo.05640.

2. Lund-Iversen, M., E. Rankin, A. Giaccia, R.Y. Miao, Q.-T. Le, Â. Helland, and O.T. Brustugun, AXL Expression and Risk of Relapse in Non-Small Cell Lung Cancer. *American Journal of Clinical Pathology*, 2012. 138(suppl_2): p. A146-A146.doi: 10.1093/ajcp/138.suppl2.219.

3. Ishikawa, M., M. Sonobe, E. Nakayama, M. Kobayashi, R. Kikuchi, J. Kitamura, et al., Higher expression of receptor tyrosine kinase Axl, and differential expression of its ligand, Gas6, predict poor survival in lung adenocarcinoma patients. *Ann Surg Oncol*, 2013. 20 Suppl 3(Suppl 3): p. S467-76.doi: 10.1245/s10434-012-2795-3.

4. Iida, S., Y. Miki, T. Suzuki, K. Mori, M. Saito, H. Niikawa, et al., Activation of AXL and antitumor effects of a monoclonal antibody to AXL in lung adenocarcinoma. *Anticancer Res*, 2014. 34(4): p. 1821-7.doi.

5. Linger, R.M., R.A. Cohen, C.T. Cummings, S. Sather, J. Migdall-Wilson, D.H. Middleton, et al., Mer or Axl receptor tyrosine kinase inhibition promotes apoptosis, blocks growth and enhances chemosensitivity of human non-small cell lung cancer. *Oncogene*, 2013. 32(29): p. 3420-31.doi: 10.1038/onc.2012.355.

6. Qu, X.H., J.L. Liu, X.W. Zhong, X.I. LiQ.G. Zhang, Insights into the roles of hnRNP A2/B1 and AXL in non-small cell lung cancer. *Oncol Lett*, 2015. 10(3): p. 1677-1685.doi: 10.3892/ol.2015.3457.

7. Sato, K., K. Suda, S. Shimizu, K. Sakai, H. Mizuuchi, K. Tomizawa, et al., Clinical, Pathological, and Molecular Features of Lung Adenocarcinomas with AXL Expression. *PLoS One*, 2016. 11(4): p. e0154186.doi: 10.1371/journal.pone.0154186.

8. Seike, M., C.H. Kim, F. Zou, R. Noro, M. Chiba, A. Ishikawa, et al., AXL and GAS6 co-expression in lung adenocarcinoma as a prognostic classifier. *Oncol Rep*, 2017. 37(6): p. 3261-3269.doi: 10.3892/or.2017.5594.

9. Wu, X., W. Ma, Q. Zhou, H. Yan, Z.F. Lim, M. Huang, et al., AXL-GAS6 expression can predict for adverse prognosis in non-small cell lung cancer with brain metastases. *J Cancer Res Clin Oncol*, 2017. 143(10): p. 1947-1957.doi: 10.1007/s00432-017-2408-4.

10. Choi, Y.J., J.H. Kim, J.K. Rho, J.S. Kim, C.M. Choi, W.S. Kim, et al., AXL and MET receptor tyrosine kinases are essential for lung cancer metastasis. *Oncol Rep*, 2017. 37(4): p. 2201-2208.doi: 10.3892/or.2017.5482.

11. Zhao, C., S. Tu, F. ZhangX. Zhang, Expression characteristics of AXL and YAP in non-small cell lung cancer and prognostic importance. *Int J Clin Exp Pathol*, 2018. 11(7): p. 3357-3365.doi.
